# SHOC2 scaffold protein modulates daunorubicin-induced cell death through p53 modulation in lymphoid leukemia cells

**DOI:** 10.1038/s41598-020-72124-1

**Published:** 2020-09-16

**Authors:** Vanessa Silva Silveira, Kleiton Silva Borges, Verena Silva Santos, Mariana Tannús Ruckert, Gabriela Maciel Vieira, Elton José Rosas Vasconcelos, Luis Fernando Peinado Nagano, Luiz Gonzaga Tone, Carlos Alberto Scrideli

**Affiliations:** 1 Department of Genetics, Ribeirão Preto Medical School, University of São Paulo, Ribeirão Preto, São Paulo Brazil; 2 Department of Pediatrics, Ribeirão Preto Medical School, University of São Paulo, Ribeirão Preto, São Paulo Brazil; 3 Leeds Omics, University of Leeds, Leeds, UK

**Keywords:** Cancer, Haematological cancer, Leukaemia, Acute lymphocytic leukaemia

## Abstract

SHOC2 scaffold protein has been mainly related to oncogenic ERK signaling through the RAS-SHOC2-PP1 phosphatase complex. In leukemic cells however, SHOC2 upregulation has been previously related to an increased 5-year event-free survival of pediatric pre-B acute lymphoid leukemia, suggesting that SHOC2 could be a potential prognostic marker. To address such paradoxical function, our study investigated how SHOC2 impact leukemic cells drug response. Our transcriptome analysis has shown that SHOC2 can modulate the DNA-damage mediated by p53. Notably, upon genetic inhibition of SHOC2 we observed a significant impairment of p53 expression, which in turn, leads to the blockage of key apoptotic molecules. To confirm the specificity of DNA-damage related modulation, several anti-leukemic drugs has been tested and we did confirm that the proposed mechanism impairs cell death upon daunorubicin-induced DNA damage of human lymphoid cells. In conclusion, our study uncovers new insights into SHOC2 function and reveals that this scaffold protein may be essential to activate a novel mechanism of p53-induced cell death in pre-B lymphoid cells.

## Introduction

SHOC2 is a scaffold protein that acts as part of the RAS-SHOC2-PP1 phosphatase complex to facilitate interactions of core signaling partners of the ERK pathway^1^. Since ERK1/2 signaling is involved in regulating several critical cellular processes, numerous studies have explored the biological significance of SHOC2 in the ERK pathway. For instance, gain-of-function germline mutations have been extensively studied in RASopathies, such as Noonan syndrome, and more recently gain-of-function somatic mutations of SHOC2 have been related to oncogenic ERK signaling in solid tumors such as lung and pancreatic cancer^2–4^. SHOC2 has also been shown to be involved in autophagy-mediated cell death through RAS-ERK-mTOR axis crosstalk by controlling key autophagy molecules such as RICTOR and RAPTOR^4^.

However, at present, a specific role for SHOC2 scaffold function in tumorigenesis and drug response has not been described. In a previous study, our group has shown that SHOC2 upregulation significantly increased the 5-year event-free survival of pediatric patients with pre-B acute lymphoid leukemia (ALL). This finding was observed both in univariate and multivariate analysis, suggesting that SHOC2 scaffold protein could be a potential prognostic marker for a favorable outcome in this cohort^5^. Since increased SHOC2 could provide oncogenic activation through ERK/MAPK signaling, this finding was unexpected and prompted us to investigate how SHOC2 absence would impact lymphoid leukemia cells. Here we show that genetic inhibition of SHOC2 promotes cell proliferation and chemoresistance when treating human lymphoid cell lines with the anthracycline daunorubicin (DNR). Furthermore, SHOC2 inhibition reduced p53 expression, leading to the blockage of key apoptotic molecules and thus, impairing cell death upon DNA damage stress. Our study uncovers new insights into SHOC2 function and reveals that SHOC2 may be essential to activate a novel mechanism of p53-induced cell death in pre-B lymphoid cells.

## Results and discussion

**Figure 1:**
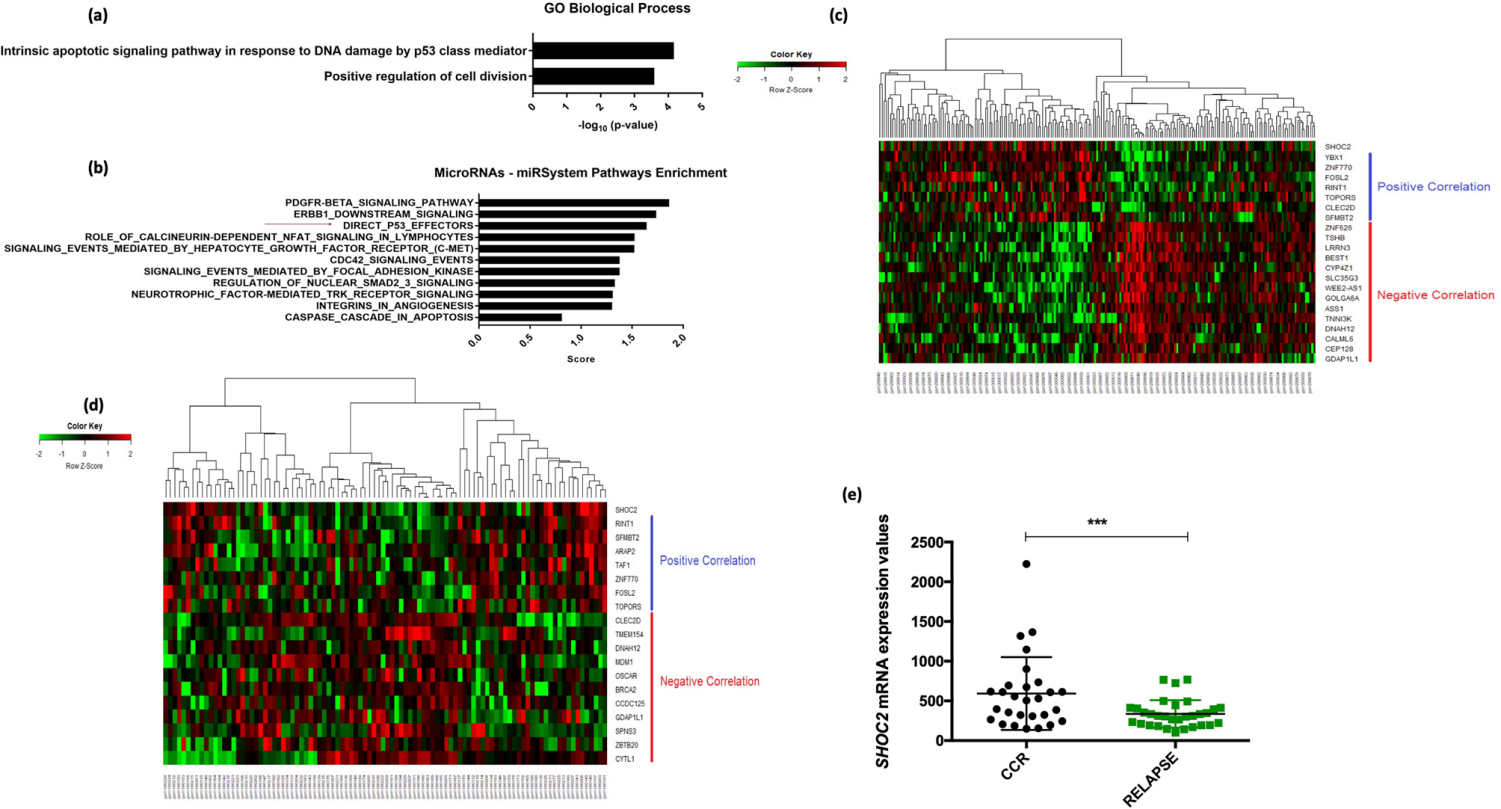
(**a**) Reh cells transcriptome profile. Enriched Gene ontology (GO) biological processes of the differentially expressed mRNA transcripts (Fold change > 5.0) between control (SCR) and SHOC2 knockdown was performed by DAVID Functional Annotation Bioinformatics Microarray Analysis tool. The most important biological process—GO:0042771 “*intrinsic apoptotic signaling pathway in response do DNA damage by p53 class-mediator*” suggest p53-related pathways, followed by GO:0051781 “*positive regulation of cell division*”. Among the top regulated genes are *TOPORS*, *VEGFA, ARAP2* and *TAF1* all p53 related genes as previously reported^6–9^. (**b**) A set of most differentially expressed miR transcripts upon SHOC2 knockdown were subjected to functional enrichment using miR-System Pathway Enrichment tool. The major class of miR transcripts predicted targets which are direct p53-effectors (total genes of the Term: 137; https://public.ndexbio.org/#/network/67c3b75d-6191-11e5-8ac5-06603eb7f303). (**c,d**) *SHOC2* expression analysis was performed using two independent public domain microarray data-set of pediatric pre-B ALL (GSE7440 and GSE11877) which showed similar patterns for SHOC2-correlated gene-sets. (**e**) *SHOC2* expression profile analysis from high-risk ALL patients regarding early response and outcome (GSE7440). *SHOC2 *upregulation was observed in patients who presented a complete clinical remission (CCR) compared to patients who relapsed (RELAPSE) (*p* < 0.001).

First, to gain insights into the underlying mechanisms of SHOC2 in pre-B ALL we examined how SHOC2 knockdown affects the mRNA and miR transcriptome profile of human B lymphoid leukemia Reh (ATCC CRL-8286) (Supplementary microarray data). To that aim a set of down-regulated and up-regulated mRNA (Fold change > 5.0) and miR transcripts were analyzed by functional analysis of biological process and for pathway enrichment. Of note, the GO enrichment analysis pointed to “intrinsic apoptotic signaling pathway in response to DNA damage by p53 class-mediator” as the most important biological process (Fig. 1a; Additional GO pathways are described in Supplementary Fig. 1a). Similarly, miR-System Pathway Enrichment predicted targets to be direct p53-effectors (Fig. 1b). Our mRNA transcriptome finding was validated in two independent public domain microarray datasets of pediatric pre-B ALL (GSE7440 and GSE11877), in which in silico analysis of the same gene-set showed a similar pattern for *SHOC2*-related gene expression (Fig. 1c,d). Additionally, we observed that *SHOC2* upregulation was significantly associated with complete clinical remission in the GSE7440 ALL datasets when compared to relapsed patients (Fig. 1e) suggesting that its expression was related to early response and a favorable outcome. Together, these data corroborate our previous findings^5^ on *SHOC2* potential as a prognostic marker and strongly suggest that SHOC2 may play a role in DNA damage-induced cell death mediated by p53-axis.

**Figure 2:**
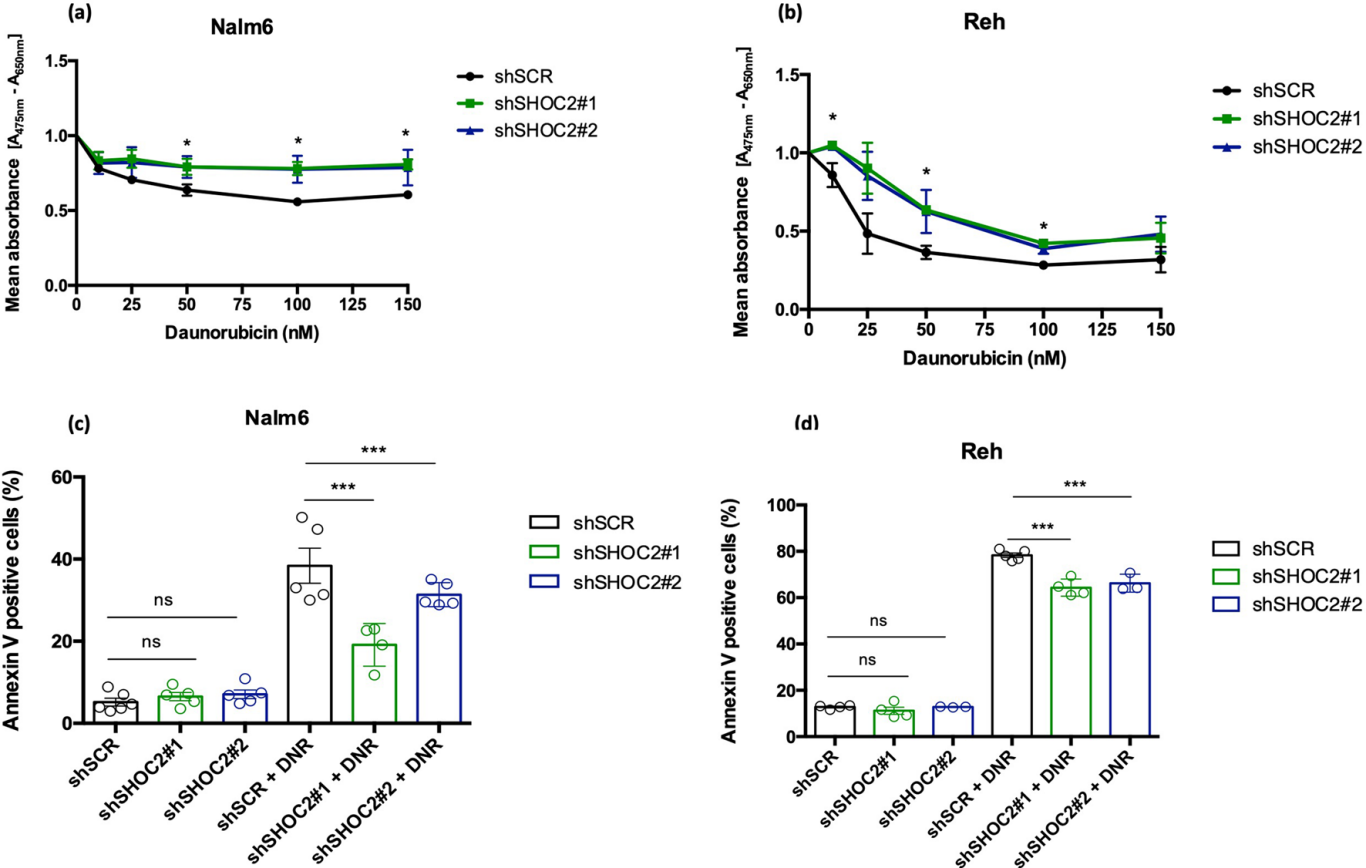
(**a**,**b**) Cell viability of Nalm-6 and Reh cells stable transduced with shRNA for *SHOC2* or SCR control were evaluated in a 4-day MTT assay after treatment with increasing concentrations of Daunorubicin (10ηM, 25ηM, 50ηM, 100ηM and 150ηM). Results are expressed as mean absorbance [A475 nm–A650 nm] with standard deviation (S.D.) from three independent experiments. Representative viability curves after 72 h of Daunorubicin exposure show how SHOC2 absence has greatly increased daunorubicin resistance (*p < 0.05). (**c**,**d**) SHOC2 inhibition cleared impaired apoptosis-induced cell death in both Reh and Nalm-6 cells (***p < 0.001) after Daunorubicin treatment for 72 h (respective IC_50_ values). The bar-graphs represent mean with S.D. from three independent experiments. Statistically significant analyses are indicated by asterisks: *p < 0.05, **p < 0.01.

In ALL treatment protocols several cytotoxic agents are used including DNR, which has been widely used in remission induction phase^10^. Proposed mechanisms for anthracycline action include the generation of toxic lesions, DNA damage, and double-strand breaks, leading to apoptosis-induced cell death mediated by p53^11^. Since our analysis of the downstream effects of SHOC2 inhibition indicated that this gene is involved in p53 mediated cell death in response to DNA damage we examined the role of SHOC2 inhibition after DNR treatment in 4-day viability assays in two pre-B acute lymphoid leukemia cell lines: Nalm-6 and Reh. DNR treatment showed that knockdown of SHOC2 in the two pre-B leukemic cell lines was associated with resistance to DNR-induced damage effects exhibiting a marked increase in the drug IC_50_ values (IC_50_ in Reh cells increased from 20.98 ± 10.94 to 84.47 ± 19.03 and in Nalm-6 cells from 123.24 ± 13.81 to 476.11 ± 25.70; *p* < 0.05 and *p* < 0.001, respectively) (Fig. 2a-b). These results suggest that SHOC2 is important for DNR efficacy. These findings are supported by previously published data that have identified SHOC2 among a set of differentially expressed genes related to DNR-sensitive B-lineage ALL^12^. To determine whether SHOC2-mediated response was specific to DNR, we tested the major anti-leukemic chemotherapy agents: Methylprednisolone, L-asparaginase and Vincristine in Reh pre-B cells (Sigma-Aldrich, Steinheim, Germany). In keeping with the idea that response to SHOC2 knockdown was specific for DNR, there was no apparent modification of the intrinsic response of the cells (Supplementary Fig. 2a-d).

**Figure 3:**
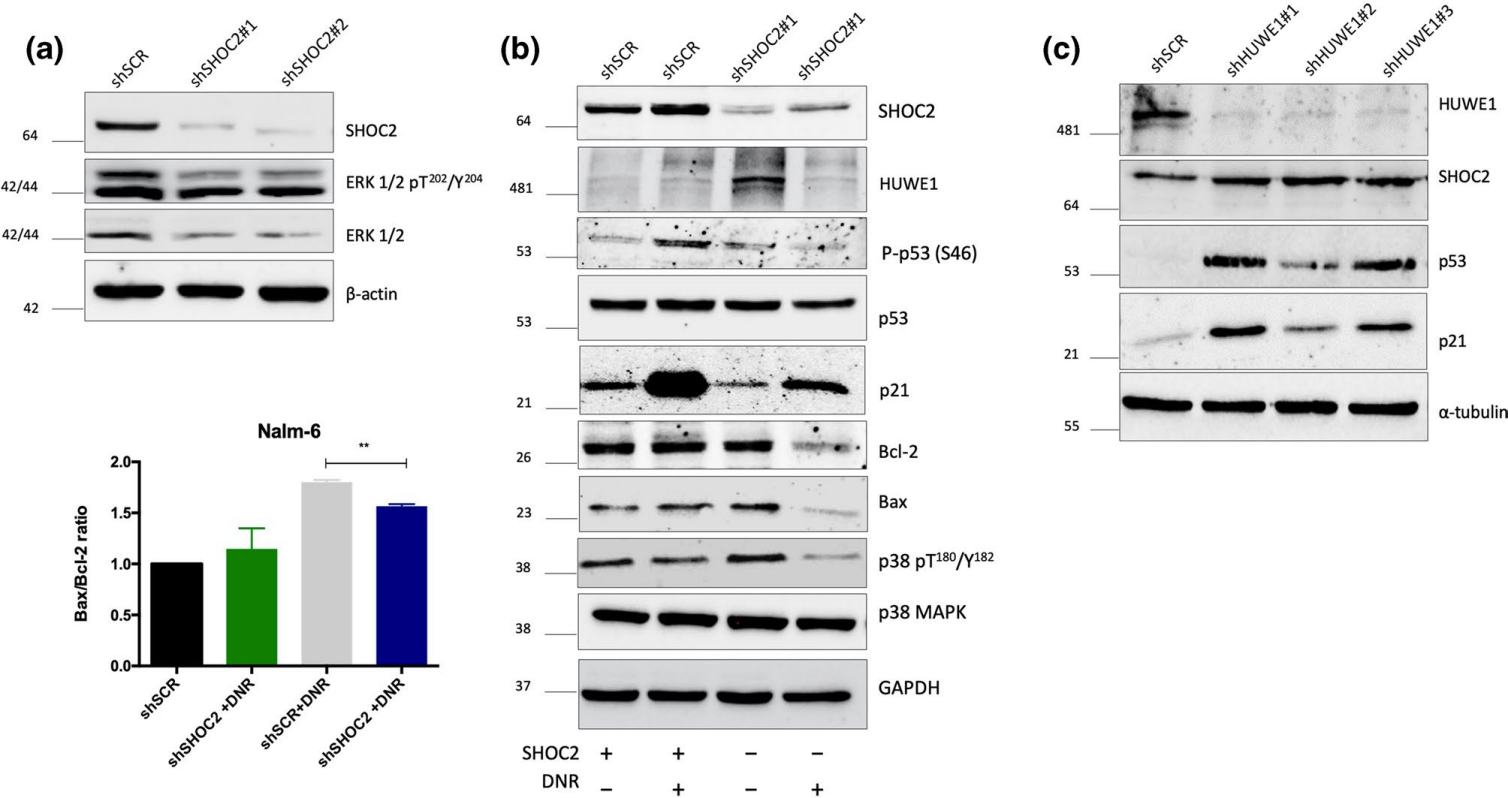
(**a**) Representative immunoblot of SHOC2 knockdown (obtained from two independent shRNA) significantly impaired ERK1/2 phosphorylation. (**b**) Combined genetic inhibition of SHOC2 with Daunorubicin treatment for 24 h (40ηM) in Nalm-6 cells. Immunoblots reveal reduced p53 expression, impaired Serine 46 phosphorylation and consequently reduced p21 expression upon Daunorubicin-induced damage. Important apoptotic effectors were also affected as observed for p38 phosphorylation inhibition and reduced BAX/BCL-2 ratio (representative bar-graph in the left panel). (**c**) HUWE1 knockdown in Nalm-6 cells greatly increased SHOC2, p53 and p21 expression, confirming SHOC2-p53 interaction. As observed in immunoblot panel in (**b**) after Daunorubicin-induced damage, in SHOC2 absence decreased p53 phosphorylation and also reduced HUWE1 expression.

To address daunorubicin-induced cell death, SHOC2-inhibited Nalm-6 and Reh cells were treated with DNR and apoptosis was measured after 72 h. DNR-induced cell death was SHOC2-dependent in both pre-B cell lines in comparison to control (shSCR) cells (Fig. 2c,d). Interestingly, in T-ALL Jurkat cells, which is p53 deficient, neither apoptosis nor cell proliferation modulation was apparent (Supplementary Fig. 3a–c). These data confirm that SHOC2 can mediate DNR effects in pre-B ALL cells and are in consonance with our transcriptome data, suggesting that SHOC2 could activate the p53 pathway. Next, we performed immunoblot analysis on pre-B Nalm-6 cells, which harbor wild type p53, to evaluate if SHOC2 inhibition has any downstream effect and, as expected, ERK1/2 phosphorylation was inhibited (Fig. 3a).

We next sought to investigate the modulation of the p53-mediated cell death cascade by SHOC2 upon DNR treatment. We found that genetic inhibition of SHOC2 combined with DNR treatment for 24 h in Nalm-6 cells significantly reduced p53 expression and impaired Serine 46 phosphorylation (Fig. 3b). This post-translation modification of p53 is known to activate damage-induced apoptosis^13^. Furthermore, we analyzed whether SHOC2 is involved in the DNA damage-mediated regulation of p21, the main target of p53 activation^14^. In agreement with previous reports investigating DNA damage responses^15^, DNA-damage induced by DNA led to the upregulation of p21 levels in wild type Nalm6. However, daunorubicin-induced p21 accumulation was significantly abolished in SHOC2 knockdown cells (Fig. 3b). Upon DNR treatment, SHOC2 knockdown impaired crucial effectors of the intrinsic apoptotic cascade such as p38 activation and reduced BAX/BCL-2 ratio (Fig. 3b right and left panel). In RAS-mutant tumors, however, SHOC2 deletion has been implicated in sustained ERK pathway suppression and consequently promotion of BIM-dependent apoptosis^16^ suggesting that SHOC2 mechanistic role in cell death could act differently in specific tumorigenic contexts. Our findings are the first to demonstrate a relationship between SHOC2 and p53-mediated cell death in leukemia response.

Previous studies have demonstrated that the SHOC2 scaffold complex interacts with the E3 ubiquitin-ligase HUWE1, which modulates ubiquitination of SHOC2 and RAF-1 utilizing a negative feedback loop that fine-tunes ERK1/2 activation^17^. HUWE1 has been reported to mediate proteasomal degradation of MYC, N-MYC, p53 and other proteins^18^. Since HUWE1 interacts with both SHOC2 and p53, we investigated whether p53-HUWE1 interaction could be modified by SHOC2. To test this hypothesis, Nalm-6 leukemic cells were transduced with shRNA lentiviral constructs targeting HUWE1 and SCR controls to evaluate SHOC2 and p53 protein expression. As observed in Fig. 3c, HUWE1 knockdown greatly increased SHOC2 expression, leading to accumulation of p53 and p21. Collectively, our findings suggest that SHOC2 plays a role in p53-HUWE1 interactions related to proteasomal degradation.

We then analyzed HUWE1 after DNR treatment and there was no difference in HUWE1 expression upon DNR-induced damage in SHOC2 knockdown cells compared to control (Fig. 3c). This result was unanticipated as we expected that without SHOC2 mediated p53 inactivation there would be HUWE1 upregulation. However, this finding does agree with previous studies that have shown that in response to genotoxic stress HUWE1 can be downregulated by self-ubiquitination and subsequent proteasomal degradation in an attempt to stabilizes p53^19^. Here we confirm that upon DNR treatment, p53 modulation through SHOC2 is a HUWE1-independent mechanism that points to alternative pathways used by SHOC2 to regulate p53 activation after DNA damage. Other mechanisms cannot be ruled out as well, considering the complexity of DNA-damage cell response.

Collectively, our results suggest that the scaffold protein SHOC2 is essential for p53 stabilization and cell death control upon daunorubicin-induced DNA damage. This report provides new and relevant insights regarding SHOC2 function in DNA damage response and its potential clinical value as a prognostic marker for patients with acute lymphoblastic leukemia undergoing daunorubicin treatment.

## Methods

### Cell culture and knockdown construction

The commercial human Leukemic cell lines Nalm-6 (DSMZ ACC128), Reh (ATCC CRL-8286) and Jurkat (ATCC TIB-152) were cultured in RPMI 1640 medium (Corning, Manassas, VA, USA) supplemented with 10% fetal bovine serum (Gibco BRL, Carlsbad, CA, USA), penicillin (100 U/mL) and streptomycin (100 μg/mL) in a humidified atmosphere with 5%CO_2_ at 37 °C, as recommended by ATCC. Cell line authentication was performed by Short Tandem Repeats (STR) DNA Profiling Analysis and all cell cultures haven been periodically tested to avoid mycoplasma contamination.

All cell lines were stably transduced with lentivirus particles containing short hairpin (sh) RNA constructs with selectable markers (GFP and/or puromycin) for *SHOC2* gene or scramble shRNA—(shSCR) (Sigma-Aldrich—Mission shRNA—SHCLNV-NM_007373 and SHC002V). For *HUWE1* knockdown cells were transfected with plasmids for lentiviral package (CMV Δ8.91 and VSV-G) and pRRL-SFFV-GFPmiRE plasmid carrying either a scrambled sequence or a shHUWE1 sequence for lentiviral package. Short hairpins sequences were the following: #1, 5′-TTAATGTTCGTAAAGAAGCTGC-3′; #2, 5′-TTCCTCTGTACCAACAACCTGC-3′; and #3, 5′- TTAAAGTCTGTACTGCTGCTGC-3′ as previously described^20^. All cell lines were transduced with lentivirus particles in RPMI 1640 medium (Corning, USA) supplemented with 10% fetal bovine serum, penicillin (100 U/mL), streptomycin (100 lg/mL) and 4 µg/ml Polybrene (Sigma-Aldrich). The culture medium was removed 48 h post-transduction and stable cells were selected either per cell sorting or puromycin (#9620, Sigma-Aldrich) selection. SHOC2 and HUWE1 knockdown was confirmed by immunoblotting (IB).

### mRNA and miR transcriptome analysis

mRNA and miR transcriptome analysis were performed as previously described^21^. Briefly, Reh cells (1 × 10^5^) transduced either with shRNA for *SHOC2* or SCR were used to RNA extraction. Total RNA was extracted using the RNeasy mini kit (Qiagen, Courtaboeuf, France) according to manufacture instructions. RNA quantification was performed in a NanoDrop Spectrophotometer (Thermoscientific, Wilmington, Delaware, USA) and RNA integrity (RIN) was analyzed on the Agilent 2100 BioAnalyzer (Santa Clara, California, USA). High quality RNA (RIN > 8.0) was amplified and labeled using the Quick Amp Labeling (one color) Kit (Agilent Technologies, Santa Clara, California, USA). The hybridization on microarrays was performed using 4 × 44K Agilent Whole Human Genome microarray slides, version 1 (Agilent AMADID 014850, Santa Clara, California, USA). Data were treated with GeneSpring 11.5 software (Agilent Technologies) and the method “Significance Analysis of Microarrays paired with permutations (100)” was applied to the identification of differentially expressed genes. A lower limit of 5.0-fold change (FC) and a maximum of 1% false discovery rate have been assigned to obtain more accurate and significant results.

MicroRNA library was obtained from total RNA (100ηg) and the expression profile was performed using the Agilent “Human micro-RNA Microarray Kit (v3)” (G4470C, Agilent Technologies, Santa Clara, CA, USA) as previously reported^21^. Spot images were processed with Feature Extraction Software v10.7.3.1 (Agilent Technologies, Santa Clara, CA, USA). Statistical analysis of microarray data regarding differential expression of miRNA were performed using the AgiMicroRna AFE package (Agilent Feature Extraction) from the Bioconductor library. For miRNA microarray analysis, signal intensity, background signal, internal control blade and spike-in controls were used. To determine the differential expression and the false discovery rate (FDR) an empirical Bayesian approach was used to control the false-positive rate. Normalization was performed by the 75% quartile method.

### Datasets processing and analysis

Two independent data-set samples were obtained from GSE7440 and GSE11877 and analyzed thought *R2 Genomic Analysis and Visualization platform (*https://r2.amc.nl*)*. We used the median values as a cut-off parameter to distinguish patients with high expression or low expression. Gene correlation analysis has been performed using Pearson’s correlation test. Additionally, we performed expression analysis to compare patients who had complete clinical remission (CCR) with patients who experienced relapse (REL) using Mann–Whitney non-parametric test.

### Drugs and functional assays

Daunorubicin (DNR #30450), Methylprednisolone (MPRED #BP248), L-Asparaginase (L-ASP #A4887) and Vincristine (VCR #V8388) were purchased from Sigma Chemical CO (Sigma-Aldrich, Steinhem, Germany). All drugs were dissolved in saline for stock solution preparation and were kept at − 20 °C. Drugs were dissolved in culture medium immediately before the experiments. For cell viability assays cells were seeded (1 × 10^4^ cells/well) in 96-well plates in complete RPMI 1640 medium, in a final volume of 200 μL. A 4-day MTT Assay (according to manufacturer’s instructions) was performed for each drug. IC_50_ values were calculated using the CalcuSyn Software (Biosoft, Cambridge, UK).

To address apoptosis, a total amount of 5 × 10^5^ cells were plated in a six-well plate and treated independently with DNR, MPRED and VCR for 72 h with the respective IC_50_ values for each cell line. Isolated cells from culture were washed and labeled with FITC Annexin-V (BD Biosciences, #556419) and propidium iodide, according to manufacturer’s instructions. Fluorescence was accessed in the BD FACSCalibur (BD Biosciences) and analysis was performed in FlowJo Software (FlowJo, LLC, 2013–2017).

### Immunoblotting

Cells were lysed in RIPA buffer on ice for 15 min. Lysates were probed with the primary antibodies: anti-SHOC2, anti-phospho-p44/42 MAPK (Erk1/2), anti-p44/42 MAPK (Erk1/2), anti-phospho-38, anti-p38, anti-phospho-p53 (Ser46), anti-p21 (#53600, #9101, #9102, #9211, #9212, #2521 and #2947 respectively, Cell Signaling Technology, Inc.); p53 (DO-1), Bax (B-9), Bcl-2 (C-2), Vinculin (N-19), β-actin (C4) and α-tubulin (B-7) (sc-126, sc-7480, sc-7382, sc-7649, sc-47778 and sc-5286, respectively; Santa Cruz Biotechnology, Santa Cruz, CA, EUA); Anti-HUWE1 (Lasu1/Ureb1, A300-486A, Bethyl Laboratories, Inc). Horseradish peroxidase (HRP)-linked secondary antibodies were used as appropriated (sc-516102; sc-2357; Santa Cruz Biotechnology). Immunoblots were quantified by densitometry using Image J 1.52a.

### Statistical analysis

Data were presented as the mean ± SD from triplicates of three independent experiments. Statistical analysis was carried out using one-way or two-way analysis of variance, followed by Bonferroni’s test, as appropriate, and Student’s *t*-test. Alpha value was set at 0.05 and significant *p* value denoted by * (*p < 0.05, **p < 0.01 and ***p < 0.001). Data analysis was carried out using the SPSS 17.0 statistical software package (SPSS Inc., Chicago, Illinois, USA).

## Supplementary information

Supplementary Figures.

Supplementary Information.
