# Supplementary material for: SHOC2 scaffold protein modulates daunorubicin-induced cell death through p53 modulation in lymphoid leukemia cells
Source: Sci Rep. 2020 Sep 16;10:15193. doi: 10.1038/s41598-020-72124-1 (PMC7495473; doi:10.1038/s41598-020-72124-1)

SHOC2 scaffold protein modulates daunorubicin-induced cell death through p53 modulation in lymphoid leukemia cells

Authors: Vanessa Silva Silveira[1*], Kleiton Silva Borges[2]; Verena Silva Santos[1]; Mariana Tannús Ruckert[1], Gabriela Maciel Vieira[1]; Elton José Rosas Vasconcelos[3]; Luis Fernando Nagano[1]; Luiz Gonzaga Tone[1,2]; Carlos Alberto Scrideli[2]

Affiliations:

1. Department of Genetics; 2. Department of Pediatrics, Ribeirão Preto Medical School, University of São Paulo, Ribeirão Preto, São Paulo, Brazil.

3. Leeds Omics, University of Leeds, United Kingdom

Supplementary information

Supplementary Figure 1

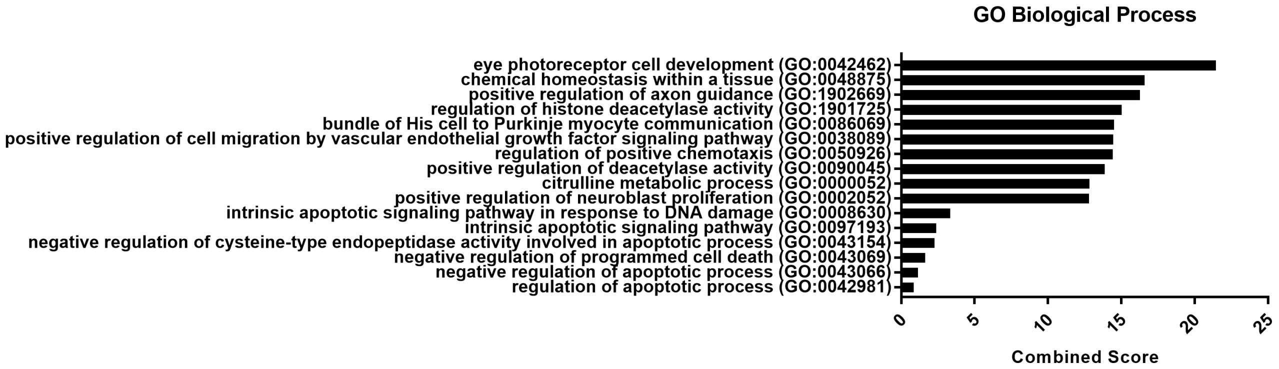

Supplementary Figure 1a. Reh cells transcriptome profile. Enriched Gene ontology (GO) biological processes of the differentially expressed mRNA transcripts between control (SCR) and SHOC2 knockdown. Top biological processes among the most relevant obtained by Enrich Functional Annotation Bioinformatics Microarray Analysis tool.

Supplementary Figure 2

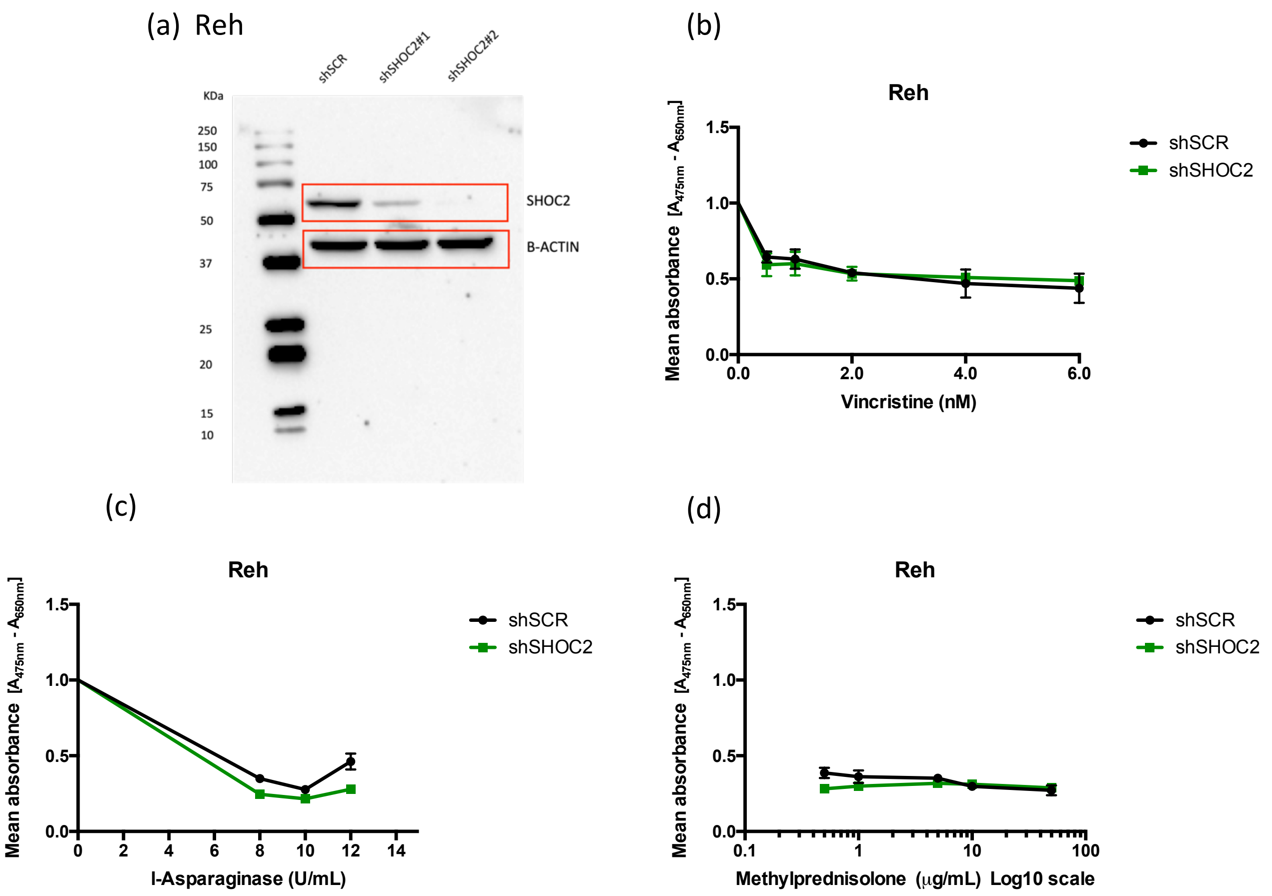

Supplementary Figure 2: (a) Representative immunoblots for SHOC2 knockdown confirmation. Uncropped images. Reh cells were transduced with lentivirus particles containing shRNA (two independent shRNA constructs) targeting *SHOC2* or scramble shRNA and stable selected with puromycin. Whole cell lysates were probed for anti-SHOC2 antibody and β-Actin was used as a loading control. Representative immunoblots from independent experiments were shown. Cell viability performed in 4-day MTT assay. Reh cells were exposed to **(b)** Vincristine (0.5 to 6.0 ηM), **(c)** L-asparaginase (8.0 to 12.0 U/mL) and **(d)** Methylprednisolone (0.5 to 50.0 μg/mL). Representative curves are presented after 72h of treatment.

Supplementary Figure 3

(a)

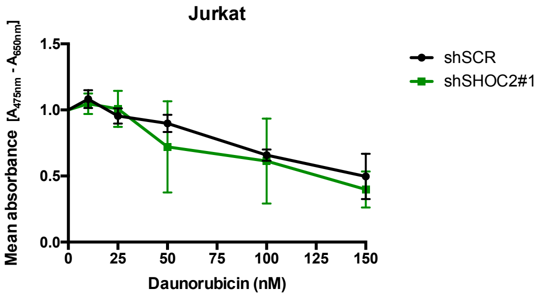

(b)

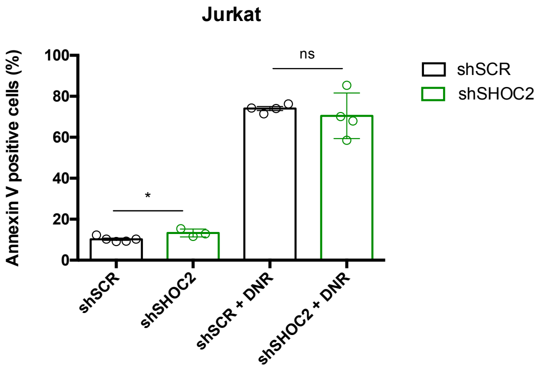

(c)

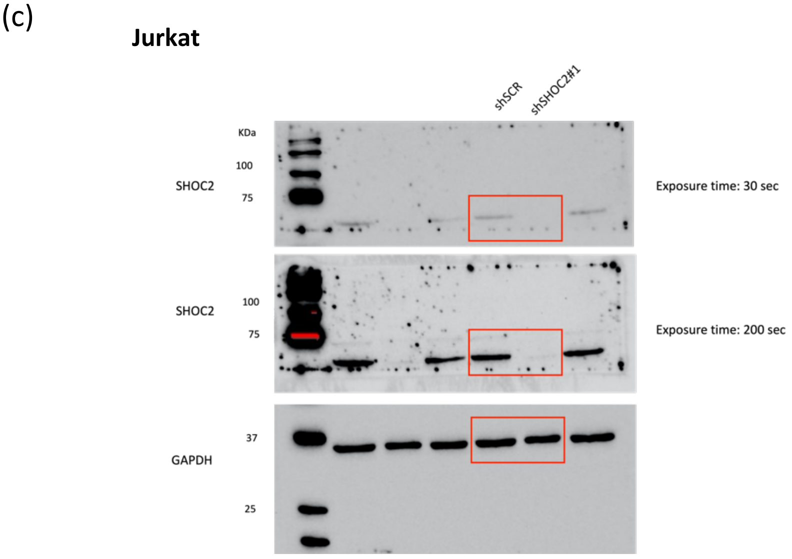

Supplementary Figure 3: In Jurkat T-ALL cells SHOC2 inhibition had no major effect after Daunorubicin treatment neither on **(a)** cell proliferation nor on apoptosis induction **(b)** (100ηM for 72h; p = 0.55). **(c)** Representative immunoblots for SHOC2 knockdown confirmation. The bar-graphs represent mean with S.D. from three independent experiments.

The uncropped images of all blots shown in the major panel of this study.

Figure 3a

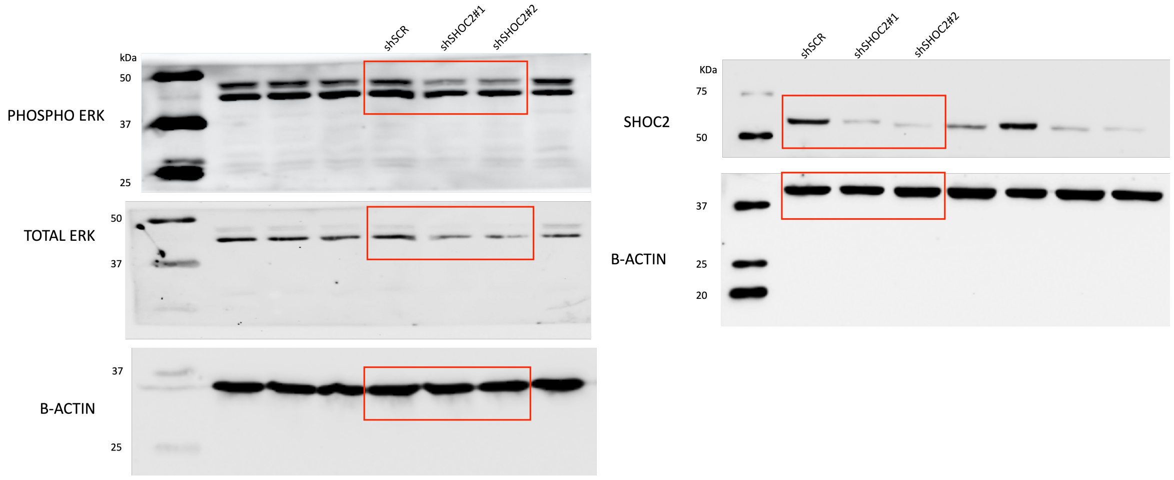

Figure 3b

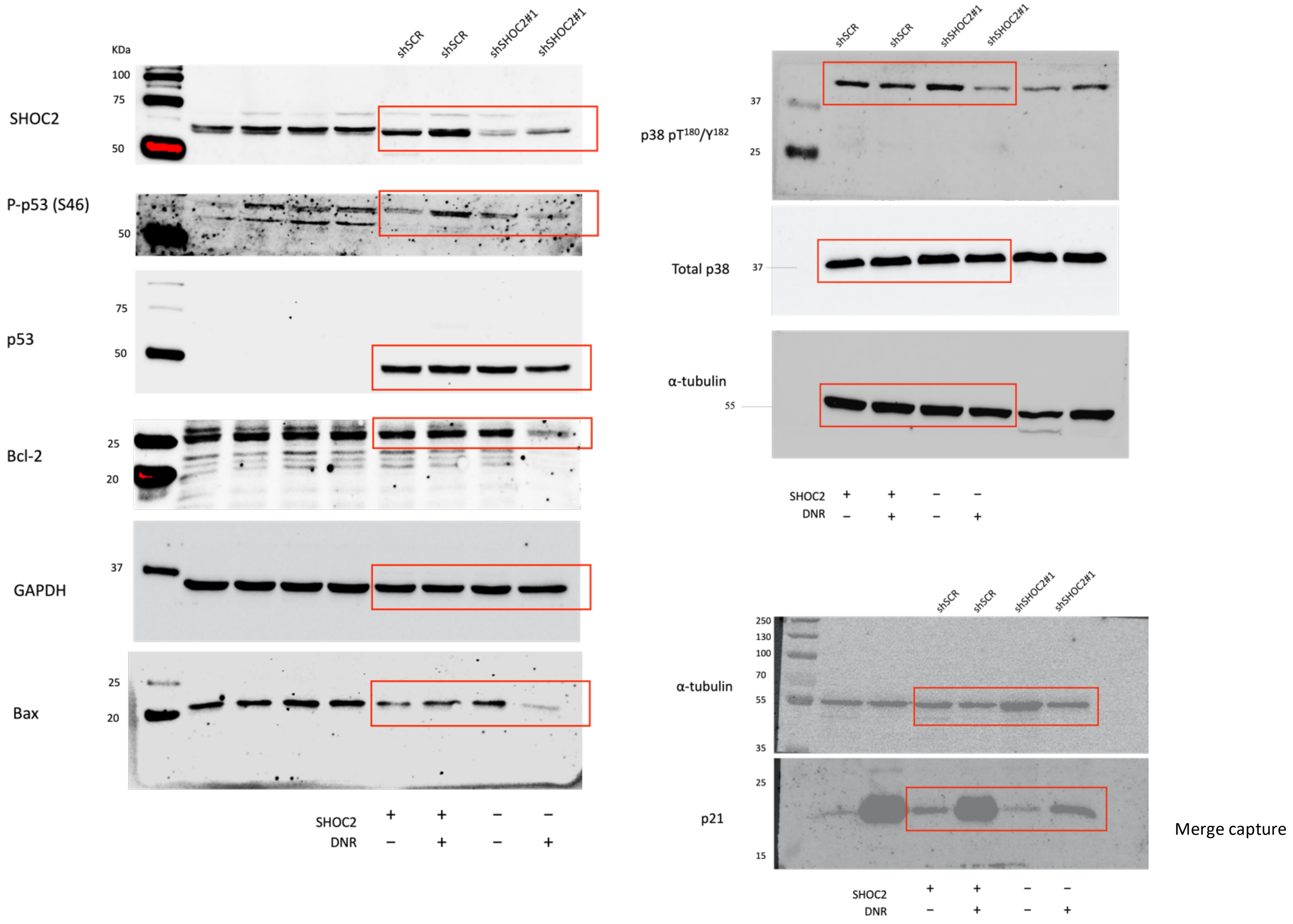

Figure 3b

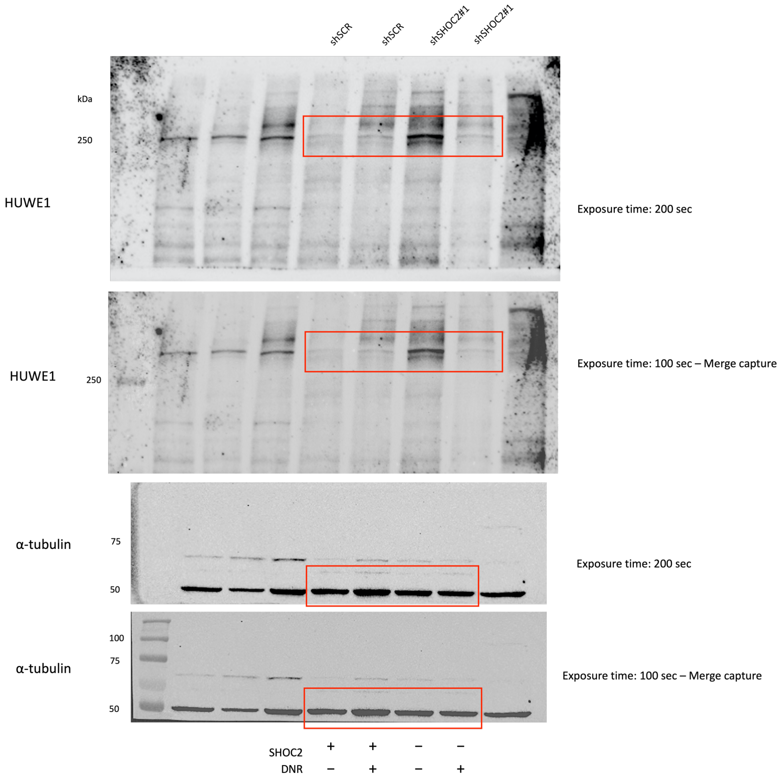

Figure 3c

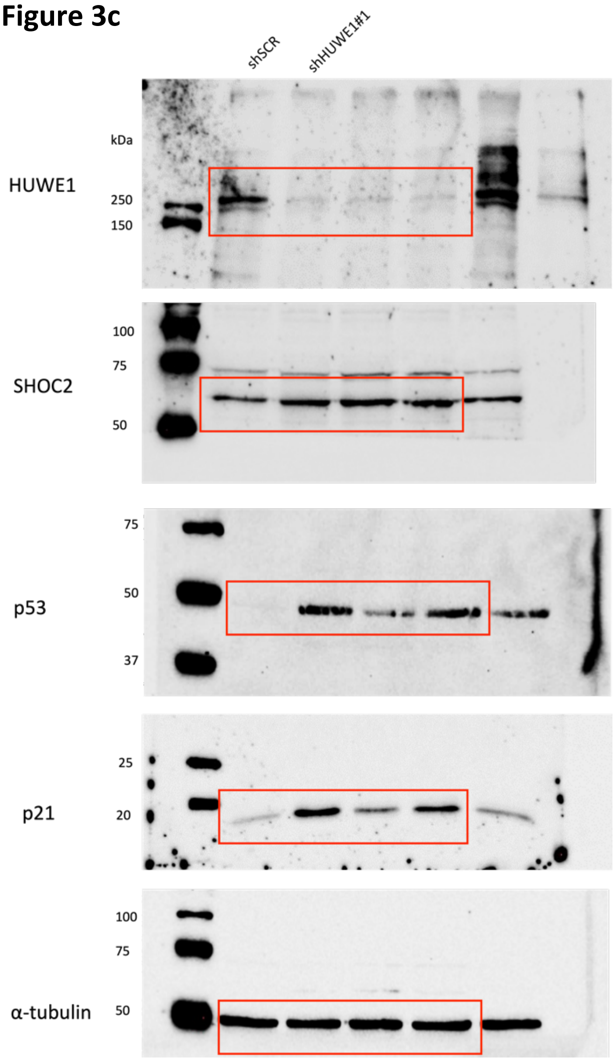

Supplement: Supplementary file 1 — Supplementary Figures. [file 41598_2020_72124_MOESM1_ESM.pdf]
