# Supplementary material for: SHOC2 scaffold protein modulates daunorubicin-induced cell death through p53 modulation in lymphoid leukemia cells
Source: Sci Rep. 2020 Sep 16;10:15193. doi: 10.1038/s41598-020-72124-1 (PMC7495473; doi:10.1038/s41598-020-72124-1)
Supplement: Supplementary file 2 — Supplementary Information. [file 41598_2020_72124_MOESM2_ESM.pdf]

SHOC2 scaffold protein modulates daunorubicin-induced cell death through p53 modulation in lymphoid leukemia cells
Silveira et al.

Reh cell line: shSCR versus shRNA for SHOC2

Current settings

Input parameters

	Two class unpaired
Data type?	
Array or Seq data?	array
Arrays centered?	FALSE
Delta	1.421122786
Minimum fold change	2
Test statistic	standard
Are data are log scale?	FALSE
Number of permutations	100
Input percentile for exchangeability factor s0	Automatic choice
Number of neighbors for KNN	10
Seed for Random number generator	94240968

Computed values

Estimate of pi0 (proportion of null genes)	0.948
s0 percentile	5.005
False Discovery Rate (%)	0.235520926

List of Significant Genes for Delta = 1.421

Negative genes (805)

Row	Gene ID	Gene Name	Score(d)	Numerator(r)	Denominator(s+s0)	Fold Change	q-value(%)
####	A_25_P00010390	hsa-miR-30b-5p	-18.84688187	-248.6713083	13.19429442	0.302466404	0
####	A_25_P00010682	hsa-miR-30d-5p	-18.58482741	-535.0113667	28.78753485	0.275913077	0
1096	A_25_P00014936	hsa-miR-1228-3p	-17.89002878	-40.51086667	2.264438318	0.396661579	0

4264	A_25_P00012212	hsa-miR-125a-3p	-15.80496834	-110.1599117	6.969954592	0.153750472	0
8475	A_25_P00010682	hsa-miR-30d-5p	-15.49777547	-540.0758167	34.84860248	0.2953873	0
5028	A_25_P00010682	hsa-miR-30d-5p	-15.19525215	-545.8779333	35.92424318	0.279183254	0
####	A_25_P00010868	hsa-miR-192-5p	-14.72950557	-61.34629167	4.164857494	0.191276086	0
####	A_25_P00013922	hsa-miR-30b-5p	-13.99790825	-972.62865	69.48385664	0.312333479	0
####	A_25_P00010683	hsa-miR-30d-5p	-13.56457309	-416.2629	30.68750466	0.293017831	0
4594	A_25_P00010683	hsa-miR-30d-5p	-13.56442558	-370.53525	27.31669305	0.29313134	0
####	A_25_P00010682	hsa-miR-30d-5p	-13.52988608	-524.255	38.74792418	0.282563244	0
9631	A_25_P00012086	hsa-miR-34a-5p	-13.40505	-62.32723	4.649533573	0.182246441	0
3323	A_25_P00013174	hsa-miR-21-3p	-13.34849863	-28.2915765	2.119457573	-0.10130793	0
2743	A_25_P00010683	hsa-miR-30d-5p	-13.22731033	-394.69425	29.83934302	0.296864436	0
####	A_25_P00010683	hsa-miR-30d-5p	-13.18608776	-412.0167833	31.24632497	0.291308685	0
9933	A_25_P00010868	hsa-miR-192-5p	-13.14354642	-74.54477333	5.671587481	0.178293584	0
9061	A_25_P00010868	hsa-miR-192-5p	-13.10358192	-70.78422833	5.401899173	0.173184829	0
9135	A_25_P00010683	hsa-miR-30d-5p	-13.05424091	-415.00355	31.79070717	0.307353556	0
####	A_25_P00010927	hsa-miR-215	-12.88965186	-48.08153667	3.730243237	0.174406965	0
1643	A_25_P00013922	hsa-miR-30b-5p	-12.72636344	-869.3759833	68.31299352	0.280164353	0
####	A_25_P00014838	hsa-miR-30b-5p	-12.72256796	-1589.59	124.9425434	0.290831431	0
####	A_25_P00010683	hsa-miR-30d-5p	-12.60983009	-411.50545	32.633703	0.298599348	0
9396	A_25_P00010682	hsa-miR-30d-5p	-12.53339659	-474.4747333	37.85683554	0.283678118	0
7619	A_25_P00012216	hsa-miR-126-3p	-12.32417098	-61.05587483	4.954156747	0.151635449	0
2040	A_25_P00013922	hsa-miR-30b-5p	-12.27147698	-826.5215167	67.35305927	0.291781841	0
4578	A_25_P00010868	hsa-miR-192-5p	-12.24838552	-64.29954	5.249633915	0.144314233	0
5069	A_25_P00010682	hsa-miR-30d-5p	-11.8923005	-482.7015167	40.58941469	0.281851552	0
####	A_25_P00010868	hsa-miR-192-5p	-11.79383374	-63.290255	5.366385215	0.241591999	0
4683	A_25_P00010682	hsa-miR-30d-5p	-11.58047515	-478.8424333	41.34911798	0.285665937	0
7306	A_25_P00010682	hsa-miR-30d-5p	-11.4960939	-503.1201333	43.76444188	0.28748185	0
7001	A_25_P00010683	hsa-miR-30d-5p	-11.37128427	-435.6650667	38.31274077	0.289855679	0
3037	A_25_P00012085	hsa-miR-34a-5p	-11.36680053	-43.72818167	3.8470088	0.193845161	0
6840	A_25_P00010390	hsa-miR-30b-5p	-11.29022336	-251.3596167	22.26347599	0.27280048	0
####	A_25_P00010683	hsa-miR-30d-5p	-11.14633363	-428.0961667	38.4069041	0.291338476	0
####	A_25_P00012086	hsa-miR-34a-5p	-11.13604935	-64.622745	5.803022506	0.185001833	0
4085	A_25_P00012216	hsa-miR-126-3p	-11.06520305	-59.48685	5.376028776	0.083334951	0
####	A_25_P00013922	hsa-miR-30b-5p	-10.7343013	-921.0706333	85.80629584	0.288785525	0
6504	A_25_P00013922	hsa-miR-30b-5p	-10.68686316	-1035.427633	96.88789106	0.288159593	0

9862	A_25_P00014838	hsa-miR-30b-5p	-10.67298258	-1710.920333	160.3038626	0.281528717	0
9557	A_25_P00012126	hsa-miR-222-3p	-10.44842404	-75.28118383	7.205027625	0.070795316	0
3652	A_25_P00010402	hsa-miR-638	-10.23321094	-409.5373833	40.02041841	0.319923412	0
8128	A_25_P00013853	hsa-miR-30a-5p	-9.97865148	-56.52769	5.664862643	0.23746199	0
####	A_25_P00010402	hsa-miR-638	-9.975248108	-395.2926167	39.62734685	0.327096179	0
####	A_25_P00012325	hsa-miR-371a-5p	-9.861382805	-76.852015	7.793229055	0.204003071	0
4872	A_25_P00011410	hsa-miR-766-3p	-9.72734938	-40.32183333	4.145202537	0.323437528	0
2400	A_25_P00010402	hsa-miR-638	-9.500002443	-387.0485333	40.7419404	0.348001662	0
####	A_25_P00012085	hsa-miR-34a-5p	-9.493339849	-47.35418083	4.988147647	0.159773728	0
7175	A_25_P00012216	hsa-miR-126-3p	-9.465196162	-70.45918067	7.444027515	0.153246256	0
1238	A_25_P00013503	hsa-miR-130b-5p	-9.455316844	-60.80881767	6.431177154	0.154074516	0
3056	A_25_P00012324	hsa-miR-371a-5p	-9.287766329	-76.56123833	8.24323477	0.228702874	0
####	A_25_P00015037	hsa-miR-320c	-9.27286674	-417.54175	45.028335	0.279480909	0
7422	A_25_P00015037	hsa-miR-320c	-9.226710113	-410.8881333	44.53246372	0.284224169	0
6871	A_25_P00012515	hsa-miR-181d	-9.192979296	-162.2100967	17.6449975	0.193839151	0
412	A_25_P00012216	hsa-miR-126-3p	-9.077289856	-54.8935435	6.047349415	0.154729054	0
6140	A_25_P00011964	hsa-miR-766-3p	-8.998294504	-48.69018667	5.411046132	0.297186085	0
1043	A_25_P00014660	hsa-miR-550a-3p	-8.957280702	-48.61921017	5.427898464	-0.02059236	0
5070	A_25_P00010868	hsa-miR-192-5p	-8.942156896	-69.94041767	7.821425913	0.093511718	0
####	A_25_P00014838	hsa-miR-30b-5p	-8.769029096	-1370.48345	156.2867947	0.29201789	0
1198	A_25_P00010390	hsa-miR-30b-5p	-8.752430026	-260.460045	29.75859781	0.264779853	0
2265	A_25_P00014182	hsa-miR-455-5p	-8.74770177	-18.73322817	2.141502838	0.082801393	0
####	A_25_P00010402	hsa-miR-638	-8.640870531	-414.4252833	47.96105691	0.316896114	0
3297	A_25_P00014838	hsa-miR-30b-5p	-8.455945389	-1560.3412	184.5259315	0.286582127	0
926	A_25_P00014953	hsa-miR-1234	-8.429080802	-31.84666667	3.778189748	0.472695722	0
6217	A_25_P00015194	hsa-miR-1268a	-8.412094147	-114.9325117	13.66277049	0.293777197	0
3424	A_25_P00012230	hsa-miR-134	-8.378564172	-263.047275	31.39526888	0.217112351	0
####	A_25_P00010402	hsa-miR-638	-8.332015922	-391.9677667	47.04356909	0.345389311	0
####	A_25_P00012324	hsa-miR-371a-5p	-8.286241033	-81.724125	9.862629469	0.204108771	0
6976	A_25_P00011998	hsa-miR-26a-5p	-8.236613182	-590.8096833	71.72968674	0.294593196	0
5590	A_25_P00011965	hsa-miR-766-3p	-8.154806999	-49.64123	6.087358046	0.333364265	0
####	A_25_P00010927	hsa-miR-215	-8.142427099	-50.46530167	6.197820509	0.179094646	0
9133	A_25_P00010927	hsa-miR-215	-7.988341924	-53.31045333	6.673531735	0.184868333	0
1087	A_25_P00010390	hsa-miR-30b-5p	-7.967719827	-222.15658	27.88207728	0.310735241	0
31	A_25_P00015037	hsa-miR-320c	-7.951917192	-399.286675	50.2126299	0.270870277	0

####	A_25_P00010642	hsa-miR-601	-7.943318293	-21.47358967	2.703352538	0.191887853	0
5289	A_25_P00015037	hsa-miR-320c	-7.8067214	-401.13475	51.38325418	0.295321558	0
8401	A_25_P00015194	hsa-miR-1268a	-7.787093866	-106.370425	13.65983598	0.35956963	0
####	A_25_P00012086	hsa-miR-34a-5p	-7.774953703	-46.41663333	5.970020543	0.23543581	0
####	A_25_P00015037	hsa-miR-320c	-7.738977766	-399.5930833	51.63383271	0.285391639	0
####	A_25_P00012212	hsa-miR-125a-3p	-7.700512877	-99.265495	12.89076411	0.198467313	0
####	A_25_P00015194	hsa-miR-1268a	-7.681218335	-117.380075	15.28143973	0.331805399	0
####	A_25_P00012212	hsa-miR-125a-3p	-7.68075983	-103.5525333	13.48206891	0.164405205	0
####	A_25_P00015194	hsa-miR-1268a	-7.668147728	-113.395915	14.78791476	0.328190617	0
####	A_25_P00012086	hsa-miR-34a-5p	-7.641803298	-58.73755167	7.686346976	0.173186676	0
2981	A_25_P00012086	hsa-miR-34a-5p	-7.629096938	-60.98198333	7.993342308	0.214645438	0
558	A_25_P00012322	hsa-miR-371a-5p	-7.618813425	-103.6234317	13.60099347	0.12007971	0
2127	A_25_P00010402	hsa-miR-638	-7.608082443	-367.4873	48.30222369	0.357727876	0
7772	A_25_P00010868	hsa-miR-192-5p	-7.602687558	-64.921005	8.53921781	0.230699258	0
####	A_25_P00010669	hsa-miR-326	-7.456344532	-23.95590717	3.212821921	0.23936212	0
####	A_25_P00014838	hsa-miR-30b-5p	-7.447723641	-1491.573133	200.2723524	0.295011583	0
####	A_25_P00015037	hsa-miR-320c	-7.429952328	-364.0956733	49.00376978	0.275881866	0
5304	A_25_P00015037	hsa-miR-320c	-7.398507017	-370.7314833	50.1089588	0.298558658	0
1757	A_25_P00015256	hsa-miR-1307-3p	-7.383618287	-29.102831	3.941540566	-0.01192111	0
####	A_25_P00012653	hsa-miR-505-3p	-7.363069576	-71.022065	9.645714232	0.165646193	0
####	A_25_P00012231	hsa-miR-134	-7.325119567	-247.957595	33.85031367	0.222359254	0
####	A_25_P00015194	hsa-miR-1268a	-7.311136425	-118.7739333	16.24561852	0.316156864	0
8772	A_25_P00013941	hsa-miR-125a-3p	-7.262597189	-97.80367167	13.46676253	0.201837677	0
5579	A_25_P00012212	hsa-miR-125a-3p	-7.18849754	-111.9994733	15.58037305	0.123248099	0
7952	A_25_P00014953	hsa-miR-1234	-7.136862671	-30.65910667	4.295880148	0.467881046	0
5619	A_25_P00010869	hsa-miR-192-5p	-7.115684795	-65.346339	9.18342238	0.11429058	0
####	A_25_P00013503	hsa-miR-130b-5p	-7.027859609	-62.24059	8.856265416	0.151934114	0
3259	A_25_P00010927	hsa-miR-215	-6.973427725	-50.38089467	7.224695896	0.112073477	0
####	A_25_P00010640	hsa-miR-601	-6.928559772	-20.39978217	2.944303411	0.170532746	0
847	A_25_P00010868	hsa-miR-192-5p	-6.912949814	-73.74301667	10.66737336	0.164907396	0
7450	A_25_P00012216	hsa-miR-126-3p	-6.845678528	-63.1962465	9.231553344	0.120350242	0
2885	A_25_P00010403	hsa-miR-638	-6.831677233	-213.1323483	31.1976607	0.300304605	0
4446	A_25_P00013174	hsa-miR-21-3p	-6.830800439	-22.94860508	3.359577737	0.076676019	0
9470	A_25_P00014661	hsa-miR-550a-3p	-6.815613204	-48.66166283	7.139733634	0.072701112	0
7855	A_25_P00015088	hsa-miR-1207-5p	-6.803851126	-1413.280683	207.7177553	0.225573882	0

5391	A_25_P00013142	hsa-miR-15a-3p	-6.79668968	-45.38403509	6.67737343	0.091575117	0
4633	A_25_P00012216	hsa-miR-126-3p	-6.763859661	-67.90872983	10.03993773	0.112919266	0
9445	A_25_P00015194	hsa-miR-1268a	-6.759754052	-104.4491517	15.45162011	0.350110265	0
####	A_25_P00012085	hsa-miR-34a-5p	-6.73207564	-45.65196883	6.781262017	0.162273928	0
3019	A_25_P00013370	hsa-miR-15b-3p	-6.714896431	-39.4965375	5.881928025	0.116596898	0
####	A_25_P00012085	hsa-miR-34a-5p	-6.704031374	-46.5296645	6.940549933	0.20535966	0
4345	A_25_P00012215	hsa-miR-126-3p	-6.684401332	-37.6603703	5.634067799	0.022827361	0
86	A_25_P00011471	hsa-miR-551a	-6.660738522	-28.31553433	4.251110329	0.152028012	0
9675	A_25_P00013484	hsa-miR-29c-5p	-6.657097923	-21.16582233	3.179436832	0.211553609	0
243	A_25_P00010926	hsa-miR-215	-6.618404551	-56.69551517	8.566341742	0.117685637	0
1592	A_25_P00011999	hsa-miR-26a-5p	-6.602169289	-1570.113117	237.8177608	0.265906992	0
4105	A_25_P00013270	hsa-miR-16-2-3p	-6.581042053	-66.36772183	10.08468284	0.159665189	0
6374	A_25_P00012459	hsa-miR-483-5p	-6.571248171	-21.70230715	3.302615665	0.062771356	0
399	A_25_P00012324	hsa-miR-371a-5p	-6.512337695	-79.89998167	12.26901697	0.193967117	0
60	A_25_P00010402	hsa-miR-638	-6.497235265	-347.96805	53.55632601	0.327352308	0
####	A_25_P00015037	hsa-miR-320c	-6.443411902	-373.8027833	58.01317517	0.280483956	0
6513	A_25_P00013087	hsa-miR-939	-6.437366983	-153.5635883	23.85503091	0.278350431	0
####	A_25_P00012216	hsa-miR-126-3p	-6.41355472	-67.2927615	10.49227214	0.119800555	0
####	A_25_P00010288	hsa-miR-181c-5p	-6.403228564	-157.9238683	24.66316277	0.22020619	0
7166	A_25_P00013853	hsa-miR-30a-5p	-6.399408118	-57.558875	8.994406036	0.263619939	0
3653	A_25_P00010390	hsa-miR-30b-5p	-6.377328013	-249.69833	39.15406727	0.286512802	0
7037	A_25_P00012230	hsa-miR-134	-6.373894024	-224.05193	35.15149909	0.197533812	0
429	A_25_P00010403	hsa-miR-638	-6.332812581	-202.6577083	32.00121679	0.31863852	0
5620	A_25_P00015004	hsa-miR-1226-5p	-6.323453865	-44.11518237	6.976437768	0.089604209	0
2848	A_25_P00010926	hsa-miR-215	-6.315751751	-46.15479733	7.307886559	0.182624733	0
995	A_25_P00012085	hsa-miR-34a-5p	-6.299152353	-43.52809667	6.910151434	0.238137775	0
7584	A_25_P00010869	hsa-miR-192-5p	-6.288196286	-54.01207667	8.589438721	0.264210481	0
2991	A_25_P00012515	hsa-miR-181d	-6.264835201	-160.6740617	25.64697339	0.214954486	0
774	A_25_P00013503	hsa-miR-130b-5p	-6.249618372	-63.27186767	10.12411701	0.134685312	0
4873	A_25_P00012085	hsa-miR-34a-5p	-6.240384758	-55.85819183	8.951081384	0.100989268	0
####	A_25_P00011096	hsa-miR-572	-6.225030744	-65.651985	10.54645153	0.381092403	0
874	A_25_P00012230	hsa-miR-134	-6.214723521	-270.2962217	43.49287957	0.203127603	0
3196	A_25_P00014660	hsa-miR-550a-3p	-6.211492784	-44.40115117	7.148225509	0.000295524	0
6929	A_25_P00011096	hsa-miR-572	-6.201616378	-74.705645	12.04615707	0.368273979	0
5695	A_25_P00015088	hsa-miR-1207-5p	-6.19729398	-1465.15965	236.4192589	0.231744301	0

1448	A_25_P00015088	hsa-miR-1207-5p	-6.164133117	-1453.373867	235.7791175	0.231244322	0
####	A_25_P00011999	hsa-miR-26a-5p	-6.125641268	-1405.995983	229.5263339	0.282460838	0
4836	A_25_P00012515	hsa-miR-181d	-6.120355414	-151.875665	24.81484403	0.200345055	0
4379	A_25_P00012357	hsa-miR-342-3p	-6.074666349	-273.75258	45.06462812	0.198096365	0
8937	A_25_P00012086	hsa-miR-34a-5p	-6.047016146	-64.62523833	10.68712846	0.150924139	0
9710	A_25_P00015087	hsa-miR-1207-5p	-6.020245046	-1403.254033	233.0891887	0.21805222	0
####	A_25_P00013545	hsa-miR-340-3p	-6.016188159	-29.4394725	4.893376291	0.192222575	0
####	A_25_P00013978	hsa-miR-296-5p	-6.011278893	-25.20904833	4.193624814	0.412797438	0
####	A_25_P00012085	hsa-miR-34a-5p	-6.008463328	-45.87637917	7.635293196	0.19733262	0
3131	A_25_P00012357	hsa-miR-342-3p	-5.987662315	-254.74478	42.54494769	0.195111295	0
1679	A_25_P00013978	hsa-miR-296-5p	-5.975015686	-23.15353	3.875057609	0.358023524	0
859	A_25_P00013243	hsa-miR-99a-3p	-5.959182974	-25.828161	4.33417821	0.018867686	0
####	A_25_P00015194	hsa-miR-1268a	-5.956982785	-104.0296217	17.46347529	0.353182225	0
7921	A_25_P00013941	hsa-miR-125a-3p	-5.912119432	-103.6832533	17.53740846	0.181931522	0
4137	A_25_P00012653	hsa-miR-505-3p	-5.893274423	-72.02805167	12.22207664	0.158652789	0
4076	A_25_P00013370	hsa-miR-15b-3p	-5.886060605	-31.676166	5.38155621	0.264558285	0
####	A_25_P00012230	hsa-miR-134	-5.862958313	-243.44319	41.52224475	0.256180747	0
5558	A_25_P00012231	hsa-miR-134	-5.853832539	-270.9597733	46.28758536	0.205309244	0
4511	A_25_P00011999	hsa-miR-26a-5p	-5.847075457	-1379.621467	235.9506863	0.286287634	0
####	A_25_P00012216	hsa-miR-126-3p	-5.833810621	-58.29160767	9.992029474	0.070696226	0
4654	A_25_P00015087	hsa-miR-1207-5p	-5.832283217	-1394.61995	239.1207522	0.206436304	0
5315	A_25_P00012261	hsa-miR-320a	-5.821484294	-128.7740267	22.12047996	0.252472321	0
4250	A_25_P00013355	hsa-miR-223-5p	-5.813671827	-28.89936383	4.9709314	-0.12092835	0
4925	A_25_P00013853	hsa-miR-30a-5p	-5.806946445	-59.25554833	10.2042526	0.202959284	0
####	A_25_P00010927	hsa-miR-215	-5.797501873	-44.165918	7.618094649	0.195189166	0
8762	A_25_P00011999	hsa-miR-26a-5p	-5.776803307	-1483.701133	256.8377448	0.275972575	0
6468	A_25_P00012215	hsa-miR-126-3p	-5.738984301	-41.23126817	7.184419055	0.009209491	0
5755	A_25_P00012231	hsa-miR-134	-5.73764411	-268.4443267	46.78650706	0.22602426	0
####	A_25_P00010836	hsa-miR-197-3p	-5.726644618	-94.93386333	16.57757198	0.260890285	0
1004	A_25_P00012231	hsa-miR-134	-5.713205795	-267.9552917	46.90103968	0.222017901	0
7371	A_25_P00010402	hsa-miR-638	-5.708145412	-372.2348667	65.21117452	0.356663161	0
6618	A_25_P00010403	hsa-miR-638	-5.690157527	-210.13836	36.93014807	0.304277367	0
5917	A_25_P00010403	hsa-miR-638	-5.671568879	-209.7290967	36.97902664	0.319771345	0
####	A_25_P00014164	hsa-miR-502-5p	-5.66858221	-16.21575267	2.860636411	0.205709256	0
9646	A_25_P00015003	hsa-miR-1226-5p	-5.656299143	-49.00966867	8.664617522	0.073920012	0

3098	A_25_P00011999	hsa-miR-26a-5p	-5.654863167	-1441.808917	254.9679584	0.284154659	0
2764	A_25_P00012230	hsa-miR-134	-5.651545219	-258.9670117	45.82233736	0.213374225	0
####	A_25_P00015256	hsa-miR-1307-3p	-5.64303972	-26.74278833	4.739074978	0.094278519	0
6361	A_25_P00014661	hsa-miR-550a-3p	-5.62039372	-47.50398513	8.452074267	0.076126223	0
6633	A_25_P00012231	hsa-miR-134	-5.614271667	-270.0822833	48.10637948	0.211373503	0
5862	A_25_P00012797	hsa-miR-548am-5p	-5.591890331	-21.82410017	3.902812622	0.134260646	0
7147	A_25_P00015088	hsa-miR-1207-5p	-5.590405962	-1493.709417	267.1915826	0.228242876	0
####	A_25_P00011410	hsa-miR-766-3p	-5.588979546	-32.62751167	5.837829857	0.412155214	0
6268	A_25_P00015088	hsa-miR-1207-5p	-5.585663245	-1473.056233	263.720917	0.235492965	0
6806	A_25_P00013087	hsa-miR-939	-5.575802582	-161.6574667	28.9926812	0.252746007	0
2055	A_25_P00012230	hsa-miR-134	-5.570548565	-264.9939467	47.57052982	0.220373221	0
####	A_25_P00011964	hsa-miR-766-3p	-5.546640444	-41.37757	7.459933705	0.362103222	0
4100	A_25_P00011998	hsa-miR-26a-5p	-5.534001332	-586.9640833	106.0650419	0.290185593	0
4214	A_25_P00012357	hsa-miR-342-3p	-5.530643182	-284.2488133	51.3952544	0.189301338	0
####	A_25_P00011998	hsa-miR-26a-5p	-5.523690134	-628.0207167	113.695863	0.287586952	0
1000	A_25_P00011998	hsa-miR-26a-5p	-5.507426772	-600.8382333	109.0960004	0.292116913	0
1743	A_25_P00015003	hsa-miR-1226-5p	-5.499457583	-46.03809733	8.371388748	0.123145212	0
####	A_25_P00013313	hsa-miR-181a-2-3p	-5.497505927	-28.29070717	5.146098529	0.052313388	0
2585	A_25_P00015087	hsa-miR-1207-5p	-5.490431347	-1385.807017	252.4040333	0.213679522	0
5879	A_25_P00013142	hsa-miR-15a-3p	-5.476215596	-36.8052446	6.72092688	0.155466848	0
6429	A_25_P00014906	hsa-miR-1224-5p	-5.467776263	-84.1788215	15.39544002	0.173661505	0
####	A_25_P00011999	hsa-miR-26a-5p	-5.45628775	-1439.578583	263.8384648	0.276271211	0
9344	A_25_P00010688	hsa-miR-498	-5.453802891	-19.6378015	3.600753803	0.21209776	0
####	A_25_P00015003	hsa-miR-1226-5p	-5.441259662	-52.680032	9.681587587	0.114304545	0
5424	A_25_P00011999	hsa-miR-26a-5p	-5.423822782	-1520.10325	280.264181	0.284758942	0
1289	A_25_P00012215	hsa-miR-126-3p	-5.379945047	-35.87315608	6.667940987	0.074996493	0
3773	A_25_P00012231	hsa-miR-134	-5.374275588	-258.77102	48.14993496	0.227342306	0
8474	A_25_P00013484	hsa-miR-29c-5p	-5.358378284	-24.50490433	4.57319417	0.143639194	0
5369	A_25_P00010926	hsa-miR-215	-5.350056394	-58.3523995	10.90687559	0.109039881	0
4623	A_25_P00015256	hsa-miR-1307-3p	-5.342757876	-27.55384783	5.157233113	-0.03158085	0
####	A_25_P00015087	hsa-miR-1207-5p	-5.316567539	-1377.367783	259.0708711	0.220316226	0
####	A_25_P00011998	hsa-miR-26a-5p	-5.299532328	-639.4782667	120.6669244	0.289304001	0
7021	A_25_P00012515	hsa-miR-181d	-5.293845265	-153.5302883	29.00165771	0.217745164	0
9650	A_25_P00012215	hsa-miR-126-3p	-5.289507777	-34.42600133	6.508356313	0.089834298	0
####	A_25_P00012126	hsa-miR-222-3p	-5.286770105	-76.4054215	14.45219292	0.069549056	0

1654	A_25_P00014907	hsa-miR-1224-5p	-5.272089675	-75.42845967	14.30712759	0.195340128	0
3161	A_25_P00012212	hsa-miR-125a-3p	-5.265153048	-100.6917467	19.12418229	0.178463593	0
####	A_25_P00012798	hsa-miR-548am-5p	-5.260485852	-24.66278967	4.688310237	0.232756165	0
5389	A_25_P00011964	hsa-miR-766-3p	-5.250203504	-51.635965	9.835040672	0.272345181	0
5866	A_25_P00015194	hsa-miR-1268a	-5.246925036	-107.60026	20.5072989	0.370160919	0
3011	A_25_P00012969	hsa-miR-708-5p	-5.235851424	-14.22847567	2.717509439	-0.41774331	0
####	A_25_P00015087	hsa-miR-1207-5p	-5.217760549	-1153.826983	221.1345217	0.212422709	0
1852	A_25_P00013270	hsa-miR-16-2-3p	-5.212254435	-58.6626695	11.25475938	0.197283346	0
155	A_25_P00012261	hsa-miR-320a	-5.207292643	-139.274885	26.74612213	0.21584181	0
584	A_25_P00011998	hsa-miR-26a-5p	-5.199674757	-595.72055	114.5688101	0.285547093	0
1028	A_25_P00014952	hsa-miR-1234	-5.199409146	-22.42425167	4.31284614	0.495169828	0
1733	A_25_P00012515	hsa-miR-181d	-5.185667479	-137.1065583	26.43951987	0.235127066	0
####	A_25_P00015087	hsa-miR-1207-5p	-5.180601811	-1351.795883	260.934141	0.214156837	0
5574	A_25_P00015004	hsa-miR-1226-5p	-5.17352657	-41.759208	8.071710358	0.074720649	0
4740	A_25_P00014907	hsa-miR-1224-5p	-5.152792514	-74.19966667	14.39989413	0.210810652	0
####	A_25_P00010288	hsa-miR-181c-5p	-5.1314907	-174.1896983	33.94524292	0.196489477	0
4545	A_25_P00012357	hsa-miR-342-3p	-5.104058333	-270.2714383	52.95226283	0.191470105	0
208	A_25_P00014851	hsa-miR-365a-3p	-5.101287268	-174.1557967	34.1395784	0.276061353	0
2966	A_25_P00015088	hsa-miR-1207-5p	-5.084607553	-1451.199983	285.4104212	0.236840075	0
####	A_25_P00010390	hsa-miR-30b-5p	-5.071669371	-244.689945	48.24643074	0.289056172	0
5081	A_25_P00010403	hsa-miR-638	-5.057287158	-210.0558717	41.53528663	0.32136334	0
9032	A_25_P00011965	hsa-miR-766-3p	-5.049830825	-43.64919667	8.643694844	0.398246118	0
828	A_25_P00012230	hsa-miR-134	-5.027501668	-255.3064	50.78196227	0.225903007	0
6885	A_25_P00013243	hsa-miR-99a-3p	-5.021799272	-27.8746776	5.550735123	0.00455899	0
6099	A_25_P00013545	hsa-miR-340-3p	-5.00638586	-41.44628097	8.278682892	0.047032	0
4291	A_25_P00014907	hsa-miR-1224-5p	-5.005269554	-74.19419033	14.82321572	0.201987962	0
5155	A_25_P00010640	hsa-miR-601	-5.004398876	-27.49463567	5.494093566	0.134137581	0
9449	A_25_P00013201	hsa-miR-26b-3p	-4.999149699	-22.08474267	4.417699809	0.153159154	0
####	A_25_P00015088	hsa-miR-1207-5p	-4.987154239	-1509.019933	302.5813642	0.227154004	0
####	A_25_P00014907	hsa-miR-1224-5p	-4.985153103	-75.41984333	15.12889209	0.211020455	0
3905	A_25_P00014906	hsa-miR-1224-5p	-4.976810802	-88.6317415	17.80894332	0.165530825	0
6350	A_25_P00013087	hsa-miR-939	-4.959119163	-150.2155917	30.29078083	0.286218437	0
1516	A_25_P00012357	hsa-miR-342-3p	-4.951392392	-259.9421283	52.49879382	0.194012969	0
470	A_25_P00010769	hsa-miR-195-5p	-4.926473017	-235.8069633	47.86527046	0.184963481	0
7890	A_25_P00012325	hsa-miR-371a-5p	-4.905948763	-72.258325	14.72871579	0.202592143	0

2481	A_25_P00012230	hsa-miR-134	-4.895973615	-261.63683	53.43918301	0.231369714	0
6107	A_25_P00012358	hsa-miR-342-3p	-4.895781638	-554.6825667	113.298061	0.187878447	0
1103	A_25_P00015087	hsa-miR-1207-5p	-4.883848918	-1376.368533	281.8204568	0.215855829	0
9702	A_25_P00010288	hsa-miR-181c-5p	-4.865571277	-152.1270583	31.26602195	0.197279829	0
4632	A_25_P00013941	hsa-miR-125a-3p	-4.861971204	-101.847275	20.94773308	0.173265298	0
####	A_25_P00013366	hsa-let-7i-3p	-4.847451209	-14.243623	2.93837367	0.093907313	0
2005	A_25_P00012358	hsa-miR-342-3p	-4.836672571	-522.4827167	108.0252403	0.190166697	0
####	A_25_P00012231	hsa-miR-134	-4.830699463	-228.7984967	47.36343017	0.195788112	0
####	A_25_P00015087	hsa-miR-1207-5p	-4.818086955	-1380.968533	286.6217539	0.21384559	0
1871	A_25_P00011002	hsa-miR-9-5p	-4.817819615	-15.91567083	3.303500775	0.169707134	0
4357	A_25_P00012418	hsa-miR-423-5p	-4.81741059	-90.24731383	18.73357318	0.167532572	0
1159	A_25_P00010770	hsa-miR-195-5p	-4.813898922	-131.98522	27.41753039	0.181416952	0
7677	A_25_P00011998	hsa-miR-26a-5p	-4.809782818	-637.9277	132.63129	0.277363801	0
####	A_25_P00012212	hsa-miR-125a-3p	-4.804217528	-99.84983667	20.7837876	0.154846866	0
1414	A_25_P00012358	hsa-miR-342-3p	-4.794331721	-509.2957133	106.2287182	0.190547256	0
####	A_25_P00012126	hsa-miR-222-3p	-4.779616911	-76.0695795	15.9154135	0.110897746	0
6266	A_25_P00012831	hsa-miR-636	-4.768559384	-15.4508745	3.240155623	-0.00938607	0
7426	A_25_P00013484	hsa-miR-29c-5p	-4.763923199	-20.21616367	4.243595629	0.175286607	0
7253	A_25_P00013941	hsa-miR-125a-3p	-4.760541234	-98.55026167	20.70148263	0.205037537	0
8796	A_25_P00010285	hsa-miR-181a-5p	-4.760014671	-2393.471383	502.8285728	0.213875399	0
3813	A_25_P00014851	hsa-miR-365a-3p	-4.749951868	-176.5203217	37.16254955	0.26473568	0
####	A_25_P00012653	hsa-miR-505-3p	-4.728037049	-71.10224217	15.03842745	0.12496254	0
9530	A_25_P00012231	hsa-miR-134	-4.723397033	-249.98509	52.92485223	0.224516993	0
8912	A_25_P00012126	hsa-miR-222-3p	-4.712082622	-75.865615	16.10023021	0.124042447	0
4505	A_25_P00010688	hsa-miR-498	-4.683679248	-24.63542333	5.259844244	0.127889212	0
7194	A_25_P00010926	hsa-miR-215	-4.661814229	-32.59352667	6.991597062	0.307353457	0
7898	A_25_P00010285	hsa-miR-181a-5p	-4.6592797	-2391.038283	513.1776663	0.212806775	0
7684	A_25_P00012357	hsa-miR-342-3p	-4.638064926	-248.7853033	53.63989235	0.205488341	0
6391	A_25_P00013086	hsa-miR-939	-4.620447982	-153.8904183	33.30638478	0.208990256	0
792	A_25_P00011998	hsa-miR-26a-5p	-4.620268971	-588.8842833	127.4567102	0.292735337	0
####	A_25_P00012953	hsa-miR-875-5p	-4.615388164	-19.53293892	4.232133511	-0.11775089	0
####	A_25_P00011410	hsa-miR-766-3p	-4.61527003	-35.420335	7.674596453	0.407807125	0
383	A_25_P00012358	hsa-miR-342-3p	-4.610495393	-470.5636283	102.063572	0.193992672	0
####	A_25_P00012828	hsa-miR-636	-4.605522592	-32.82516533	7.127348672	0.229430949	0
####	A_25_P00012358	hsa-miR-342-3p	-4.600172512	-550.5486167	119.6799936	0.201579467	0

2048	A_25_P00014662	hsa-miR-550a-3p	-4.593247439	-46.24448467	10.06792804	0.088289208	0
####	A_25_P00013277	hsa-miR-129-1-3p	-4.589054733	-23.90629833	5.209416694	0.475710596	0
####	A_25_P00015088	hsa-miR-1207-5p	-4.585411795	-1367.187367	298.160215	0.206276216	0
1983	A_25_P00015004	hsa-miR-1226-5p	-4.583059373	-38.328068	8.362987445	0.110736455	0
####	A_25_P00013642	hsa-miR-625-3p	-4.581044138	-10.0411485	2.1918908	0.278881583	0
3041	A_25_P00015003	hsa-miR-1226-5p	-4.572754745	-45.08055767	9.858512031	0.123801012	0
4030	A_25_P00012409	hsa-miR-345-5p	-4.571259688	-30.310885	6.630751055	0.041986838	0
####	A_25_P00013201	hsa-miR-26b-3p	-4.570226417	-22.70502133	4.968029866	0.103918234	0
6623	A_25_P00012357	hsa-miR-342-3p	-4.568757792	-267.5974183	58.57115446	0.176970858	0
####	A_25_P00013853	hsa-miR-30a-5p	-4.56737947	-58.30827833	12.76624347	0.251878688	0
1923	A_25_P00010869	hsa-miR-192-5p	-4.561859445	-61.8858035	13.56591632	0.151470363	0
4307	A_25_P00010869	hsa-miR-192-5p	-4.556270692	-60.70897833	13.32426944	0.166241716	0
####	A_25_P00011096	hsa-miR-572	-4.543318657	-67.40121167	14.83523758	0.384935014	0
9767	A_25_P00010403	hsa-miR-638	-4.541871899	-205.550515	45.2567839	0.310928024	0
3210	A_25_P00012952	hsa-miR-875-5p	-4.53955376	-13.68894187	3.015481828	-0.0956205	0
####	A_25_P00012086	hsa-miR-34a-5p	-4.536836338	-45.99694	10.13854955	0.370526558	0
2487	A_25_P00014953	hsa-miR-1234	-4.523620518	-31.917055	7.055643786	0.472478418	0
9883	A_25_P00012653	hsa-miR-505-3p	-4.516824361	-70.748058	15.66322982	0.123349771	0
218	A_25_P00011999	hsa-miR-26a-5p	-4.506507777	-1650.706567	366.2939572	0.268070773	0
9256	A_25_P00010287	hsa-miR-181c-5p	-4.506030681	-42.52271649	9.436845752	0.03927254	0
####	A_25_P00010344	hsa-miR-557	-4.497317628	-15.833133	3.520572552	0.269182112	0
4640	A_25_P00015256	hsa-miR-1307-3p	-4.49477225	-33.14591833	7.374326548	-0.05279604	0
####	A_25_P00010288	hsa-miR-181c-5p	-4.493743794	-152.3834583	33.91013492	0.202458664	0
4906	A_25_P00011411	hsa-miR-766-3p	-4.489254342	-38.0063255	8.466066435	-0.02857544	0
592	A_25_P00012358	hsa-miR-342-3p	-4.474553645	-493.0778	110.1959746	0.192552943	0
####	A_25_P00010770	hsa-miR-195-5p	-4.464407858	-113.9811617	25.53108168	0.175878545	0
3168	A_25_P00013546	hsa-miR-340-3p	-4.450163108	-53.53324767	12.02950237	0.20352679	0
2717	A_25_P00013086	hsa-miR-939	-4.436697056	-140.534105	31.67538897	0.246935934	0
5621	A_25_P00011702	hcmv-miR-US4	-4.426562909	-14.9691449	3.381663202	-0.1181693	0
####	A_25_P00010288	hsa-miR-181c-5p	-4.408178649	-189.2934933	42.94142965	0.190298533	0
3608	A_25_P00012085	hsa-miR-34a-5p	-4.408167737	-38.0179565	8.624435087	0.225104923	0
####	A_25_P00013545	hsa-miR-340-3p	-4.407625592	-37.35167783	8.474330918	0.001044791	0
####	A_25_P00013607	hsa-miR-505-5p	-4.405133622	-24.29114433	5.514280932	-0.00300686	0
####	A_25_P00010285	hsa-miR-181a-5p	-4.402113094	-2183.98445	496.1218404	0.204674229	0
####	A_25_P00010288	hsa-miR-181c-5p	-4.399366909	-154.1167783	35.03158103	0.220766749	0

1199	A_25_P00010926	hsa-miR-215	-4.396361749	-35.6523345	8.109508847	0.214999367	0
5395	A_25_P00013545	hsa-miR-340-3p	-4.394588371	-39.61134703	9.013664919	0.053391952	0
7595	A_25_P00011965	hsa-miR-766-3p	-4.390758743	-56.08047667	12.77238854	0.301375101	0
7348	A_25_P00012418	hsa-miR-423-5p	-4.369136625	-88.6664525	20.29381549	0.178134019	0
4167	A_25_P00012215	hsa-miR-126-3p	-4.356211276	-38.75894633	8.897398192	0.034421397	0
4605	A_25_P00013087	hsa-miR-939	-4.340963654	-152.1475467	35.04925606	0.282234075	0
8055	A_25_P00010285	hsa-miR-181a-5p	-4.329983995	-2421.94145	559.3418943	0.212247137	0
9911	A_25_P00010600	hsa-miR-126-5p	-4.329125529	-11.529381	2.663212449	0.261653217	0
7094	A_25_P00013853	hsa-miR-30a-5p	-4.327438925	-60.43323333	13.96512681	0.237973559	0
####	A_25_P00014851	hsa-miR-365a-3p	-4.323070835	-160.125905	37.03985225	0.294707972	0
4816	A_25_P00012358	hsa-miR-342-3p	-4.31667744	-466.8061383	108.1401482	0.195385781	0
3614	A_25_P00012126	hsa-miR-222-3p	-4.312128603	-68.734891	15.93989821	0.118259718	0
####	A_25_P00010285	hsa-miR-181a-5p	-4.311837469	-2297.921033	532.9331288	0.210853383	0
####	A_25_P00014907	hsa-miR-1224-5p	-4.311199338	-73.37089333	17.01867336	0.235381146	0
6314	A_25_P00012323	hsa-miR-371a-5p	-4.302822943	-80.92035167	18.8063401	0.167785389	0
####	A_25_P00012325	hsa-miR-371a-5p	-4.29465213	-74.07708167	17.24868032	0.21980209	0
1010	A_25_P00013853	hsa-miR-30a-5p	-4.286965068	-62.84679667	14.65997405	0.217827335	0
3017	A_25_P00010869	hsa-miR-192-5p	-4.275457605	-61.229264	14.32110189	0.152645561	0
####	A_25_P00013545	hsa-miR-340-3p	-4.272825045	-27.5073615	6.437745803	0.144825824	0
9653	A_25_P00012418	hsa-miR-423-5p	-4.271444894	-88.58858867	20.73972412	0.169146966	0
7988	A_25_P00012261	hsa-miR-320a	-4.270437801	-134.5852217	31.515556	0.28180907	0
####	A_25_P00010926	hsa-miR-215	-4.269301001	-42.1098335	9.863402344	0.183577146	0
631	A_25_P00010927	hsa-miR-215	-4.268022787	-54.68861267	12.81357092	0.129546115	0
####	A_25_P00010927	hsa-miR-215	-4.261472064	-43.16984	10.13026469	0.242362434	0
####	A_25_P00013243	hsa-miR-99a-3p	-4.253581175	-25.4412523	5.98113713	0.047144822	0
7639	A_25_P00010927	hsa-miR-215	-4.240383032	-48.64292333	11.47135128	0.207386107	0
8374	A_25_P00012357	hsa-miR-342-3p	-4.238896267	-272.2239517	64.2204797	0.209568954	0
854	A_25_P00013502	hsa-miR-130b-5p	-4.23600372	-38.52092837	9.093695596	0.056202805	0
2003	A_25_P00010287	hsa-miR-181c-5p	-4.22404018	-36.50815117	8.642945997	0.145313348	0
3354	A_25_P00012323	hsa-miR-371a-5p	-4.222135669	-82.42029667	19.52099675	0.174653819	0
8756	A_25_P00012213	hsa-miR-125a-3p	-4.198041907	-106.488695	25.36627727	0.222058943	0
875	A_25_P00010285	hsa-miR-181a-5p	-4.188915092	-2165.251933	516.9004112	0.221262468	0
####	A_25_P00013370	hsa-miR-15b-3p	-4.181911152	-32.91966	7.871917601	0.212506351	0
####	A_25_P00011854	ebv-miR-BART13	-4.161545042	-96.71507117	23.24018368	0.135767046	0
5700	A_25_P00014851	hsa-miR-365a-3p	-4.154959216	-182.3819417	43.89500166	0.268894703	0

1286	A_25_P00010761	hsa-miR-27b-3p	-4.137645037	-107.575145	25.99912367	0.20551288	0
1570	A_25_P00010285	hsa-miR-181a-5p	-4.132890832	-2217.602533	536.5741859	0.209184847	0
8169	A_25_P00010643	hsa-miR-601	-4.123913232	-29.8217	7.231408209	0.107695226	0
####	A_25_P00010640	hsa-miR-601	-4.115281346	-20.9912455	5.100804474	0.258357888	0
5648	A_25_P00013608	hsa-miR-505-5p	-4.111663049	-24.94848117	6.067734848	-0.05713873	0
5955	A_25_P00010770	hsa-miR-195-5p	-4.11120761	-127.7569017	31.07527369	0.179009692	0
1573	A_25_P00014181	hsa-miR-455-5p	-4.110174887	-12.11749866	2.948171061	0.015865914	0
3099	A_25_P00012261	hsa-miR-320a	-4.108575149	-123.4672367	30.05110827	0.268333919	0
####	A_25_P00012261	hsa-miR-320a	-4.098135426	-127.370605	31.08013566	0.261465552	0
2027	A_25_P00014063	hsa-miR-486-5p	-4.097624536	-13.04536638	3.183641222	-0.11018316	0
8233	A_25_P00012215	hsa-miR-126-3p	-4.094705143	-39.175366	9.567322831	0.142238497	0
6697	A_25_P00013853	hsa-miR-30a-5p	-4.092376351	-59.23584833	14.47468249	0.253649507	0
3606	A_25_P00013313	hsa-miR-181a-2-3p	-4.084098938	-29.47717267	7.217546174	-0.03273488	0
4000	A_25_P00012409	hsa-miR-345-5p	-4.080058231	-32.64359983	8.000768123	-0.00089766	0
1468	A_25_P00011964	hsa-miR-766-3p	-4.078122309	-41.72916	10.23244445	0.368775495	0
4579	A_25_P00010403	hsa-miR-638	-4.071641927	-165.239675	40.58305665	0.327248504	0
9295	A_25_P00012971	hsa-miR-708-5p	-4.06876368	-17.20586515	4.228769843	0.094894518	0
####	A_25_P00012515	hsa-miR-181d	-4.068521477	-144.4831117	35.51243676	0.22362676	0
9076	A_25_P00013546	hsa-miR-340-3p	-4.068479734	-52.1226045	12.81132214	0.120780022	0
8093	A_25_P00012358	hsa-miR-342-3p	-4.038327238	-467.37104	115.7338206	0.188845289	0
1340	A_25_P00010288	hsa-miR-181c-5p	-4.021074346	-145.6021467	36.2097624	0.215945298	0
650	A_25_P00010285	hsa-miR-181a-5p	-4.009590697	-2029.472583	506.1545521	0.214540613	0
####	A_25_P00013209	hsa-miR-29a-5p	-4.005551704	-31.34801783	7.826142352	0.119552786	0
5654	A_25_P00015122	hsa-miR-1299	-3.995079424	-14.8446505	3.715733512	-0.27586419	0
####	A_25_P00014906	hsa-miR-1224-5p	-3.990680431	-79.43574833	19.90531432	0.197427735	0
6838	A_25_P00012952	hsa-miR-875-5p	-3.979682966	-17.807762	4.474668498	-0.11762747	0
5546	A_25_P00011096	hsa-miR-572	-3.977852045	-70.13001333	17.63012112	0.368883001	0
2044	A_25_P00013087	hsa-miR-939	-3.949705431	-141.01898	35.70367017	0.324813176	0
####	A_25_P00011965	hsa-miR-766-3p	-3.943060115	-55.374525	14.04354065	0.306445807	0
####	A_25_P00013086	hsa-miR-939	-3.937620278	-138.2485683	35.10967503	0.2631633	0
4487	A_25_P00011854	ebv-miR-BART13	-3.911019967	-101.7958483	26.02795414	0.119203341	0
####	A_25_P00013978	hsa-miR-296-5p	-3.890282092	-22.07364667	5.674047831	0.426440973	0
8506	A_25_P00010869	hsa-miR-192-5p	-3.881685048	-62.09498333	15.99691437	0.147602669	0
####	A_25_P00013174	hsa-miR-21-3p	-3.880146578	-22.12062667	5.700977069	-0.03177757	0
####	A_25_P00010770	hsa-miR-195-5p	-3.878021557	-136.88233	35.29694923	0.198053931	0

4994	A_25_P00013449	hsa-miR-149-3p	-3.877312208	-17.177618	4.430290129	-0.18104768	0
####	A_25_P00013021	hsa-miR-760	-3.875730305	-20.59492533	5.313817967	-0.02305125	0
6761	A_25_P00013489	hsa-miR-30c-1-3p	-3.874692291	-22.9644745	5.926786639	0.163058625	0
####	A_25_P00010635	hsa-miR-584-5p	-3.870823555	-18.00280083	4.650896787	0.228396168	0
9776	A_25_P00010815	hsa-miR-30c-5p	-3.869668668	-179.2121083	46.31200336	0.216752319	0
####	A_25_P00010688	hsa-miR-498	-3.866802415	-14.93970117	3.863580179	0.256947793	0
3919	A_25_P00013853	hsa-miR-30a-5p	-3.863427294	-62.28229833	16.12099662	0.226338889	0
7702	A_25_P00012653	hsa-miR-505-3p	-3.861805861	-73.33638067	18.99017799	0.130938874	0
2106	A_25_P00014837	hsa-miR-27b-3p	-3.852201735	-170.3138833	44.21208832	0.213818964	0
2112	A_25_P00012647	hsa-miR-502-3p	-3.848606403	-22.24150208	5.779105409	-0.15398296	0
1029	A_25_P00013545	hsa-miR-340-3p	-3.844593822	-37.91963152	9.863104733	0.079628115	0
####	A_25_P00011854	ebv-miR-BART13	-3.843116793	-95.62565167	24.88231735	0.170446763	0
####	A_25_P00011096	hsa-miR-572	-3.84122484	-56.721875	14.76661153	0.375685917	0
####	A_25_P00012515	hsa-miR-181d	-3.822649144	-160.0058933	41.85733174	0.22495276	0
1365	A_25_P00014837	hsa-miR-27b-3p	-3.816654857	-174.98425	45.84754361	0.240237805	0
####	A_25_P00015094	hsa-miR-548e	-3.804767295	-11.52724767	3.029685332	-0.12656726	0
6049	A_25_P00014897	hsa-miR-582-5p	-3.804342514	-13.09257417	3.441481443	-0.17454775	0
3870	A_25_P00013277	hsa-miR-129-1-3p	-3.803975644	-27.75422	7.296108755	0.485209393	0
802	A_25_P00012705	hsa-miR-545-3p	-3.803564581	-16.05318517	4.220563323	-0.07473291	0
975	A_25_P00012195	hsa-miR-152	-3.801636243	-17.969429	4.726761807	-0.43531695	0
920	A_25_P00012706	hsa-miR-545-3p	-3.796300325	-16.95603243	4.46646234	-0.12686955	0
1544	A_25_P00010459	hsa-miR-660-5p	-3.78902097	-170.2395583	44.92969548	0.160021106	0
6776	A_25_P00010288	hsa-miR-181c-5p	-3.787345151	-148.7721217	39.28137409	0.214007301	0
1307	A_25_P00013484	hsa-miR-29c-5p	-3.772443348	-19.60469833	5.196817163	0.190377395	0
2564	A_25_P00014837	hsa-miR-27b-3p	-3.764973827	-187.1091767	49.69733795	0.220985629	0
####	A_25_P00012653	hsa-miR-505-3p	-3.760045814	-67.855335	18.04641176	0.17030197	0
5603	A_25_P00010815	hsa-miR-30c-5p	-3.758298166	-191.3296317	50.90858234	0.19833483	0
####	A_25_P00011096	hsa-miR-572	-3.748000853	-63.93235	17.05772024	0.420649528	0
9731	A_25_P00013881	hsa-miR-129-5p	-3.745876879	-10.00882833	2.671958705	0.198701627	0
5599	A_25_P00012213	hsa-miR-125a-3p	-3.745074976	-103.9099817	27.74576806	0.218158644	0
1462	A_25_P00013021	hsa-miR-760	-3.743437363	-28.23711267	7.543097408	-0.43216178	0
4261	A_25_P00013086	hsa-miR-939	-3.739383331	-134.3580067	35.93052511	0.262701215	0
3374	A_25_P00010769	hsa-miR-195-5p	-3.731354483	-241.59935	64.74843145	0.180756439	0
1440	A_25_P00014906	hsa-miR-1224-5p	-3.726324874	-82.14013183	22.04320198	0.178593288	0
2178	A_25_P00014182	hsa-miR-455-5p	-3.724477155	-27.56660368	7.401469398	0.022391991	0

9235	A_25_P00010770	hsa-miR-195-5p	-3.72318422	-121.1751517	32.54610691	0.174709477	0
3147	A_25_P00013142	hsa-miR-15a-3p	-3.722510673	-40.84762833	10.97313935	0.131259064	0
6285	A_25_P00013276	hsa-miR-129-1-3p	-3.721905746	-14.76620483	3.967377424	0.227329217	0
6753	A_25_P00013607	hsa-miR-505-5p	-3.718314907	-24.0227725	6.460661107	-0.00875917	0
8355	A_25_P00012653	hsa-miR-505-3p	-3.718285824	-68.55054083	18.43606008	0.154120419	0
980	A_25_P00014837	hsa-miR-27b-3p	-3.71706333	-166.9531167	44.91532746	0.210562223	0
4824	A_25_P00012423	hsa-miR-423-3p	-3.716084951	-10.22976	2.752832654	-0.54860743	0
####	A_25_P00010769	hsa-miR-195-5p	-3.714818073	-236.8572733	63.76012732	0.186330474	0
1313	A_25_P00010869	hsa-miR-192-5p	-3.711507619	-59.99940417	16.16577691	0.152113392	0
####	A_25_P00013502	hsa-miR-130b-5p	-3.711378178	-38.4325499	10.35533111	0.0799425	0
9420	A_25_P00014907	hsa-miR-1224-5p	-3.708758582	-76.88882467	20.7316877	0.192180735	0
####	A_25_P00012325	hsa-miR-371a-5p	-3.704675902	-67.47503833	18.21347943	0.228132325	0
1858	A_25_P00013690	kshv-miR-K12-3	-3.703854684	-418.1832333	112.9048705	0.257712576	0
8742	A_25_P00013271	hsa-miR-16-2-3p	-3.698815584	-169.792145	45.90446351	0.215169357	0
####	A_25_P00013683	hsa-miR-374b-3p	-3.697525785	-12.67990973	3.429295823	0.034259919	0
6756	A_25_P00014851	hsa-miR-365a-3p	-3.69470233	-188.40275	50.9926736	0.260799565	0
3745	A_25_P00013461	hsa-miR-186-3p	-3.68209044	-13.301049	3.612363471	-0.19637183	0
9901	A_25_P00010881	hsa-miR-23b-3p	-3.681483267	-134.1216617	36.4314196	0.262248429	0
1142	A_25_P00012828	hsa-miR-636	-3.678227745	-31.89892333	8.67236222	0.301166303	0
7198	A_25_P00010770	hsa-miR-195-5p	-3.677641637	-117.65017	31.99065641	0.208624401	0
4054	A_25_P00012511	hsa-miR-193b-3p	-3.674095833	-29.40017217	8.002015599	0.242882697	0
####	A_25_P00013690	kshv-miR-K12-3	-3.66707817	-429.7288333	117.1856212	0.25156116	0
7333	A_25_P00011007	hsa-miR-194-5p	-3.659218225	-64.09414667	17.5158033	0.214912713	0
####	A_25_P00012407	hsa-miR-345-5p	-3.652535366	-9.26736395	2.537241401	0.147670206	0
9898	A_25_P00012419	hsa-miR-423-5p	-3.649741094	-192.3591217	52.70486774	0.164987173	0
####	A_25_P00010770	hsa-miR-195-5p	-3.647250833	-116.8697583	32.04324673	0.191117657	0
8398	A_25_P00012954	hsa-miR-875-5p	-3.646794287	-25.1666935	6.901045554	0.010246747	0
61	A_25_P00011411	hsa-miR-766-3p	-3.639417653	-37.633275	10.34046614	-0.05191076	0
1081	A_25_P00010578	hsa-miR-151a-3p	-3.637253052	-55.16647833	15.16707184	0.313583259	0
5376	A_25_P00013277	hsa-miR-129-1-3p	-3.636429613	-32.43412167	8.919221631	0.414732544	0
7774	A_25_P00011799	hsv1-miR-H1_v14.0	-3.633566407	-14.02179967	3.85896337	-0.0816163	0
####	A_25_P00012512	hsa-miR-193b-3p	-3.629292165	-127.6996617	35.18583125	0.31818957	0
4636	A_25_P00012459	hsa-miR-483-5p	-3.626138101	-27.80366567	7.667569435	0.003931123	0
203	A_25_P00012257	hsa-miR-193a-3p	-3.623559252	-34.99940625	9.658847507	0.047149315	0
####	A_25_P00012262	hsa-miR-320a	-3.619162228	-241.9043367	66.83987106	0.276708858	0

2302	A_25_P00013020	hsa-miR-760	-3.614472413	-24.43464175	6.760223612	-0.08002092	0
1861	A_25_P00010600	hsa-miR-126-5p	-3.610449048	-15.09543533	4.181040954	-0.09626935	0
9696	A_25_P00012213	hsa-miR-125a-3p	-3.60976127	-103.8993217	28.78287895	0.232280968	0
####	A_25_P00010769	hsa-miR-195-5p	-3.603302408	-235.993215	65.49359123	0.199593222	0
####	A_25_P00013546	hsa-miR-340-3p	-3.601569025	-65.8433175	18.28184245	0.093684196	0
9285	A_25_P00011096	hsa-miR-572	-3.599713666	-67.13789333	18.65089825	0.405861156	0
1744	A_25_P00015142	hsa-miR-1246	-3.599463068	-70.49920167	19.58603278	0.391940755	0
9534	A_25_P00013086	hsa-miR-939	-3.594338877	-135.4391917	37.68125274	0.257050674	0
4310	A_25_P00012262	hsa-miR-320a	-3.592317668	-256.75379	71.4730193	0.287425854	0
5794	A_25_P00012834	hsa-miR-652-3p	-3.591957722	-54.25904767	15.10570331	0.173564532	0
####	A_25_P00014662	hsa-miR-550a-3p	-3.589010896	-37.88723267	10.55645518	0.187915468	0
5630	A_25_P00010926	hsa-miR-215	-3.583770958	-44.05540017	12.29302896	0.164244069	0
2442	A_25_P00010761	hsa-miR-27b-3p	-3.583071808	-108.6663267	30.32769994	0.212766542	0
7049	A_25_P00013370	hsa-miR-15b-3p	-3.579735394	-32.50494467	9.080264624	0.21469644	0
314	A_25_P00010769	hsa-miR-195-5p	-3.573981048	-242.36532	67.81382351	0.181627336	0
4726	A_25_P00014851	hsa-miR-365a-3p	-3.571856916	-168.58515	47.19818122	0.254987845	0
####	A_25_P00011854	ebv-miR-BART13	-3.568201894	-96.13530333	26.94222641	0.149560544	0
6018	A_25_P00012512	hsa-miR-193b-3p	-3.566859531	-154.29047	43.25667121	0.325391717	0
1996	A_25_P00013690	kshv-miR-K12-3	-3.564562878	-427.0052667	119.7917616	0.263101842	0
####	A_25_P00010669	hsa-miR-326	-3.560220869	-24.91265817	6.997503549	0.186722405	0
9820	A_25_P00014182	hsa-miR-455-5p	-3.556708557	-11.94313977	3.357919148	0.073542252	0
8473	A_25_P00014837	hsa-miR-27b-3p	-3.555090642	-200.0725583	56.27776575	0.205253389	0
####	A_25_P00013683	hsa-miR-374b-3p	-3.553704674	-17.04422883	4.796186065	0.12217283	0
2428	A_25_P00010687	hsa-miR-498	-3.551492526	-20.59571867	5.799172747	0.223987196	0
####	A_25_P00013489	hsa-miR-30c-1-3p	-3.54430111	-17.7459235	5.006889356	0.291754912	0
####	A_25_P00013201	hsa-miR-26b-3p	-3.543606536	-17.28431773	4.877606348	0.155927207	0
7146	A_25_P00013277	hsa-miR-129-1-3p	-3.542295214	-27.25386167	7.693842557	0.488041242	0
####	A_25_P00013086	hsa-miR-939	-3.540860053	-142.2348167	40.16956743	0.246396801	0
####	A_25_P00015256	hsa-miR-1307-3p	-3.534115145	-22.54262067	6.37857561	-0.03094459	0
2859	A_25_P00012215	hsa-miR-126-3p	-3.53356802	-34.93660717	9.887062303	0.029247914	0
9880	A_25_P00010869	hsa-miR-192-5p	-3.531007145	-61.73457067	17.4835587	0.154091357	0
9874	A_25_P00015270	hsa-miR-320d	-3.524532318	-297.8064633	84.49531356	0.283481331	0
8409	A_25_P00013502	hsa-miR-130b-5p	-3.522517865	-33.64823151	9.552323878	0.044821159	0
3908	A_25_P00011853	ebv-miR-BART13	-3.517204971	-168.549455	47.92141953	0.141714758	0
####	A_25_P00012953	hsa-miR-875-5p	-3.512739546	-12.5761031	3.580141065	0.085759298	0.1840722

1006	A_25_P00010642	hsa-miR-601	-3.508383363	-30.5253455	8.700686995	0.141531022	0.1840722
2204	A_25_P00012512	hsa-miR-193b-3p	-3.5058575	-123.7874	35.30873688	0.321669761	0.1840722
317	A_25_P00015003	hsa-miR-1226-5p	-3.502679273	-42.07664967	12.0127041	0.046606202	0.1840722
####	A_25_P00010815	hsa-miR-30c-5p	-3.500800755	-190.0619167	54.29098369	0.182041313	0.1840722
####	A_25_P00010769	hsa-miR-195-5p	-3.499308446	-238.41337	68.13156761	0.207182807	0.1840722
1706	A_25_P00012419	hsa-miR-423-5p	-3.497078682	-186.207765	53.24666155	0.164143123	0.1840722
6371	A_25_P00010475	hsa-miR-100-5p	-3.493728795	-21.07959133	6.033551135	-0.08742981	0.1840722
####	A_25_P00012213	hsa-miR-125a-3p	-3.492517929	-103.57493	29.6562343	0.252987691	0.1840722
2648	A_25_P00012834	hsa-miR-652-3p	-3.489090587	-56.21872533	16.11271589	0.191252633	0.1840722
####	A_25_P00012196	hsa-miR-152	-3.480662609	-79.059715	22.71398406	0.159220505	0.1840722
####	A_25_P00012512	hsa-miR-193b-3p	-3.478716991	-158.0701117	45.43919844	0.292052945	0.1840722
####	A_25_P00012262	hsa-miR-320a	-3.475018175	-280.1574067	80.62041479	0.260192434	0.1840722
####	A_25_P00010881	hsa-miR-23b-3p	-3.472809066	-131.208995	37.78180502	0.261091623	0.1840722
####	A_25_P00010815	hsa-miR-30c-5p	-3.468408154	-177.0923	51.05866788	0.221923212	0.1840722
3667	A_25_P00010853	hsa-miR-212-3p	-3.465885891	-35.64080033	10.28331614	0.239497657	0.1840722
2285	A_25_P00012086	hsa-miR-34a-5p	-3.45997164	-42.036471	12.14936866	0.244821572	0.1840722
####	A_25_P00013978	hsa-miR-296-5p	-3.458832891	-24.09530667	6.966311304	0.415193356	0.1840722
####	A_25_P00012418	hsa-miR-423-5p	-3.458416713	-89.8226915	25.97220027	0.179461581	0.1840722
####	A_25_P00014837	hsa-miR-27b-3p	-3.450784678	-165.1743383	47.86573309	0.232636454	0.1840722
####	A_25_P00012721	hsa-miR-570-3p	-3.446937663	-8.36816045	2.427708671	-0.11083963	0.1840722
5965	A_25_P00014852	hsa-miR-365a-3p	-3.44585706	-178.20291	51.71511961	0.270575573	0.1840722
7076	A_25_P00013087	hsa-miR-939	-3.443066035	-139.9616117	40.65028386	0.305992342	0.1840722
9878	A_25_P00012952	hsa-miR-875-5p	-3.439590857	-22.36379782	6.501877329	-0.03831894	0.1840722
5539	A_25_P00012262	hsa-miR-320a	-3.438352546	-258.5711717	75.20205336	0.289478339	0.1840722
####	A_25_P00010770	hsa-miR-195-5p	-3.436142503	-127.5822317	37.12949377	0.19568371	0.1840722
4989	A_25_P00010403	hsa-miR-638	-3.432661838	-162.3157567	47.28568217	0.345288376	0.1840722
####	A_25_P00013086	hsa-miR-939	-3.42380219	-130.8205167	38.209134	0.26237864	0.1840722
4277	A_25_P00011651	ebv-miR-BHRF1-1	-3.412923981	-10.30052217	3.018093056	-0.47457055	0.1840722
####	A_25_P00015035	hsa-miR-320b	-3.412302497	-326.9738383	95.82205524	0.286635842	0.1840722
4734	A_25_P00015270	hsa-miR-320d	-3.410375614	-262.2660317	76.90238887	0.285067298	0.1840722
2383	A_25_P00012418	hsa-miR-423-5p	-3.408269548	-81.93799067	24.04093618	0.198901612	0.1840722
8089	A_25_P00014852	hsa-miR-365a-3p	-3.405766463	-177.64058	52.15876718	0.269269819	0.1840722
####	A_25_P00014907	hsa-miR-1224-5p	-3.405044902	-71.188552	20.90678803	0.197977406	0.1840722
####	A_25_P00013087	hsa-miR-939	-3.402410379	-137.6477233	40.45594388	0.324216192	0.1840722
2826	A_25_P00015003	hsa-miR-1226-5p	-3.396587094	-44.6907111	13.15753427	0.097822229	0.1840722

2680	A_25_P00015034	hsa-miR-320b	-3.395204788	-168.3625833	49.5883441	0.268040748	0.1840722
####	A_25_P00012126	hsa-miR-222-3p	-3.393650157	-72.28155867	21.29906011	0.096601873	0.1840722
3727	A_25_P00013086	hsa-miR-939	-3.392799271	-140.2031867	41.32374935	0.241040822	0.1840722
####	A_25_P00010815	hsa-miR-30c-5p	-3.39196158	-175.4729617	51.73200154	0.201779427	0.1840722
3927	A_25_P00012262	hsa-miR-320a	-3.386829274	-236.6089183	69.86148376	0.282429511	0.1840722
3389	A_25_P00013484	hsa-miR-29c-5p	-3.379830475	-27.14281017	8.030819997	0.177616866	0.1840722
6926	A_25_P00014662	hsa-miR-550a-3p	-3.379475067	-51.677229	15.29149586	0.129862537	0.1840722
3657	A_25_P00012838	hsa-miR-548d-5p	-3.373280676	-35.82265543	10.61953003	0.103865764	0.1840722
####	A_25_P00013690	kshv-miR-K12-3	-3.37265615	-426.5997333	126.4877635	0.258524096	0.1840722
####	A_25_P00012419	hsa-miR-423-5p	-3.37122897	-188.40014	55.88470605	0.165338445	0.1840722
8179	A_25_P00012419	hsa-miR-423-5p	-3.368194842	-188.07346	55.83805831	0.178666868	0.1840722
2010	A_25_P00012261	hsa-miR-320a	-3.363603538	-123.9129667	36.83934961	0.272928851	0.1840722
4209	A_25_P00013382	hsa-miR-30b-3p	-3.363342736	-21.46193283	6.381131665	0.191291066	0.1840722
####	A_25_P00010769	hsa-miR-195-5p	-3.356190638	-236.2017533	70.37793105	0.193503667	0.1840722
7726	A_25_P00010769	hsa-miR-195-5p	-3.354348932	-257.2377367	76.68782881	0.204082214	0.1840722
####	A_25_P00010881	hsa-miR-23b-3p	-3.353250661	-154.467425	46.06498011	0.254496527	0.1840722
####	A_25_P00015142	hsa-miR-1246	-3.348621431	-78.70355333	23.50326991	0.37752522	0.1840722
####	A_25_P00013546	hsa-miR-340-3p	-3.347820455	-57.179779	17.07970298	0.140703778	0.1840722
447	A_25_P00012838	hsa-miR-548d-5p	-3.342122293	-27.49343683	8.226340756	0.138511168	0.1840722
####	A_25_P00013365	hsa-let-7i-3p	-3.341867261	-13.48001375	4.033677192	-0.17532928	0.1840722
959	A_25_P00010459	hsa-miR-660-5p	-3.339797345	-177.2301267	53.06613198	0.13702065	0.1840722
3754	A_25_P00012419	hsa-miR-423-5p	-3.338128342	-193.5760067	57.98938412	0.161070796	0.1840722
4489	A_25_P00011854	ebv-miR-BART13	-3.336199336	-94.59540667	28.354243	0.144435135	0.1840722
####	A_25_P00012261	hsa-miR-320a	-3.33615016	-116.23865	34.84215171	0.277729207	0.1840722
69	A_25_P00013690	kshv-miR-K12-3	-3.331637325	-450.86125	135.3272298	0.231246226	0.1840722
7417	A_25_P00012459	hsa-miR-483-5p	-3.331508932	-25.5593516	7.672004524	0.109920887	0.1840722
####	A_25_P00014906	hsa-miR-1224-5p	-3.328531625	-75.22945867	22.6013952	0.22401087	0.1840722
9460	A_25_P00015034	hsa-miR-320b	-3.327365332	-170.784285	51.3271817	0.274749908	0.1840722
8094	A_25_P00013607	hsa-miR-505-5p	-3.32730193	-24.92884367	7.492209662	-0.10099975	0.1840722
8308	A_25_P00015271	hsa-miR-320d	-3.326888077	-414.1254467	124.4783224	0.300397436	0.1840722
706	A_25_P00012459	hsa-miR-483-5p	-3.32660463	-22.14262933	6.65622513	0.016793517	0.1840722
4933	A_25_P00012262	hsa-miR-320a	-3.326406801	-251.5825683	75.63193061	0.288654339	0.1840722
9198	A_25_P00010881	hsa-miR-23b-3p	-3.315717813	-151.991595	45.83972568	0.256533597	0.1840722
8113	A_25_P00012262	hsa-miR-320a	-3.315560427	-249.8342433	75.35204042	0.282954891	0.1840722
7696	A_25_P00013690	kshv-miR-K12-3	-3.313093287	-415.8440667	125.5153509	0.264294593	0.1840722

####	A_25_P00014837	hsa-miR-27b-3p	-3.311314879	-172.1968383	52.00255627	0.222884216	0.1840722
8016	A_25_P00012161	hsa-miR-128	-3.310791551	-1267.589983	382.8661406	0.245869243	0.1840722
8236	A_25_P00012633	hsa-miR-500a-5p	-3.309580917	-28.246219	8.534681491	0.174564323	0.1840722
9345	A_25_P00012243	hsa-miR-186-5p	-3.309093241	-189.28234	57.20066684	0.12968635	0.1840722
3764	A_25_P00013690	kshv-miR-K12-3	-3.30613189	-404.6928333	122.406742	0.270517648	0.1840722
8819	A_25_P00012834	hsa-miR-652-3p	-3.301151952	-52.78742843	15.99060849	0.127338348	0.1840722
####	A_25_P00012243	hsa-miR-186-5p	-3.298898092	-178.0125967	53.96122938	0.146526152	0.1840722
5694	A_25_P00012942	hsa-miR-892b	-3.292066825	-13.492943	4.098623666	0.376716405	0.1840722
####	A_25_P00012213	hsa-miR-125a-3p	-3.289811431	-100.21284	30.46157572	0.232695774	0.1840722
####	A_25_P00012322	hsa-miR-371a-5p	-3.288085286	-90.36889167	27.48374321	0.145970561	0.1840722
7332	A_25_P00014832	hsa-miR-181a-5p	-3.28689244	-5018.942833	1526.956822	0.194199816	0.1840722
####	A_25_P00012162	hsa-miR-128	-3.280408278	-1965.878583	599.2786314	0.251983324	0.1840722
2903	A_25_P00014861	hsa-miR-483-5p	-3.274046501	-26.03318867	7.951380243	0.237746477	0.1840722
####	A_25_P00014851	hsa-miR-365a-3p	-3.272915658	-174.5839983	53.34204011	0.286016438	0.1840722
1252	A_25_P00010287	hsa-miR-181c-5p	-3.271979124	-32.01584567	9.784856338	0.179330168	0.1840722
5929	A_25_P00012512	hsa-miR-193b-3p	-3.268867403	-140.0638117	42.84781069	0.365889287	0.1840722
####	A_25_P00015271	hsa-miR-320d	-3.268590111	-326.5410633	99.90272633	0.304815014	0.1840722
3759	A_25_P00012515	hsa-miR-181d	-3.268062677	-148.1495633	45.33253428	0.19318243	0.1840722
3110	A_25_P00014852	hsa-miR-365a-3p	-3.266501757	-164.148545	50.25209144	0.283501376	0.1840722
2780	A_25_P00013690	kshv-miR-K12-3	-3.26599933	-410.1261167	125.5744644	0.265837125	0.1840722
####	A_25_P00015035	hsa-miR-320b	-3.263572898	-286.2318	87.70504258	0.302884646	0.1840722
3180	A_25_P00010634	hsa-miR-584-5p	-3.262617755	-19.44697467	5.960543382	0.261686262	0.1840722
2278	A_25_P00012254	hsa-miR-193a-5p	-3.261556011	-17.55879318	5.383563282	-0.23884637	0.1840722
8283	A_25_P00014662	hsa-miR-550a-3p	-3.257523382	-43.94677	13.49085328	0.136645049	0.1840722
2974	A_25_P00013893	hsa-miR-199b-5p	-3.256590115	-81.24566717	24.94807891	0.078074301	0.1840722
####	A_25_P00014907	hsa-miR-1224-5p	-3.255163771	-72.9443605	22.40881431	0.18209296	0.1840722
####	A_25_P00012653	hsa-miR-505-3p	-3.254535594	-70.07931367	21.53281525	0.156280905	0.1840722
####	A_25_P00010459	hsa-miR-660-5p	-3.253723067	-180.447545	55.45879023	0.154445302	0.1840722
####	A_25_P00013087	hsa-miR-939	-3.251949386	-145.0946967	44.61775982	0.314107866	0.1840722
4909	A_25_P00012323	hsa-miR-371a-5p	-3.249825237	-67.716475	20.83695893	0.247389009	0.1840722
####	A_25_P00012860	hsa-miR-671-5p	-3.249702995	-17.99310283	5.536845324	0.217090837	0.1840722
655	A_25_P00013270	hsa-miR-16-2-3p	-3.247092531	-50.44697183	15.53604382	0.225113406	0.1840722
####	A_25_P00015270	hsa-miR-320d	-3.246311298	-290.9763883	89.63292846	0.290044754	0.1840722
####	A_25_P00012322	hsa-miR-371a-5p	-3.246134323	-93.517335	28.80883096	0.158143724	0.1840722
####	A_25_P00012418	hsa-miR-423-5p	-3.245975904	-92.5892005	28.52430309	0.147750139	0.1840722

3889	A_25_P00015271	hsa-miR-320d	-3.244363915	-365.0406233	112.5153136	0.309422851	0.1840722
####	A_25_P00011853	ebv-miR-BART13	-3.24061923	-163.8458167	50.56003345	0.158932458	0.1840722
9529	A_25_P00014851	hsa-miR-365a-3p	-3.236483354	-166.2306317	51.3614975	0.293855187	0.1840722
2343	A_25_P00015035	hsa-miR-320b	-3.232508614	-334.079805	103.3500123	0.293391713	0.1840722
1561	A_25_P00013089	hsa-miR-940	-3.232013248	-56.59596667	17.51105652	0.468030856	0.1840722
5986	A_25_P00011854	ebv-miR-BART13	-3.230332672	-95.26174917	29.48976432	0.124947163	0.1840722
####	A_25_P00010882	hsa-miR-23b-3p	-3.229803496	-53.21469167	16.4761391	0.293952315	0.1840722
1912	A_25_P00010635	hsa-miR-584-5p	-3.225875326	-15.14130833	4.693705367	0.366886583	0.1840722
####	A_25_P00010475	hsa-miR-100-5p	-3.225817013	-21.74405233	6.740634153	-0.05132442	0.1840722
2703	A_25_P00012262	hsa-miR-320a	-3.225426206	-229.9010367	71.27772331	0.291545517	0.1840722
####	A_25_P00011854	ebv-miR-BART13	-3.222265689	-92.92412667	28.83813305	0.150698604	0.1840722
8512	A_25_P00012162	hsa-miR-128	-3.218590261	-1935.586667	601.3771589	0.246458096	0.1840722
####	A_25_P00014161	hsa-miR-501-5p	-3.217857992	-9.176440667	2.851723317	0.391219771	0.1840722
4692	A_25_P00010882	hsa-miR-23b-3p	-3.214362373	-56.05611667	17.43926482	0.247184682	0.1840722
9192	A_25_P00013941	hsa-miR-125a-3p	-3.214150293	-86.57031667	26.93412217	0.213569547	0.1840722
1779	A_25_P00014924	hsa-miR-1225-3p	-3.21396072	-23.28341667	7.244462112	0.397458396	0.1840722
338	A_25_P00011471	hsa-miR-551a	-3.211964088	-19.288102	6.005080215	0.257494519	0.1840722
####	A_25_P00011700	hcmv-miR-US4	-3.211787636	-11.28733965	3.514348061	-0.15444354	0.1840722
####	A_25_P00014832	hsa-miR-181a-5p	-3.211129527	-4666.645833	1453.272375	0.192184145	0.1840722
6352	A_25_P00014832	hsa-miR-181a-5p	-3.210621377	-4915.3745	1530.972956	0.195542631	0.1840722
####	A_25_P00012418	hsa-miR-423-5p	-3.207637484	-79.76388517	24.86686403	0.185149856	0.1840722
6909	A_25_P00012162	hsa-miR-128	-3.206228786	-2314.862133	721.9890682	0.253291316	0.1840722
3483	A_25_P00012305	hsa-miR-361-3p	-3.204336459	-49.364975	15.40567778	0.174088829	0.1840722
3224	A_25_P00013090	hsa-miR-940	-3.204130698	-168.084305	52.45862946	0.347628733	0.1840722
####	A_25_P00010882	hsa-miR-23b-3p	-3.20170845	-54.54201667	17.03528523	0.248567534	0.1840722
####	A_25_P00012161	hsa-miR-128	-3.200517441	-1189.730267	371.7305994	0.254128603	0.1840722
####	A_25_P00015271	hsa-miR-320d	-3.196661426	-361.1443983	112.9754923	0.300288695	0.1840722
####	A_25_P00014852	hsa-miR-365a-3p	-3.195659547	-172.6396417	54.02316458	0.283874809	0.1840722
####	A_25_P00013503	hsa-miR-130b-5p	-3.19205391	-58.94595717	18.46646668	0.119363088	0.1840722
8082	A_25_P00012354	hsa-miR-342-5p	-3.191512201	-11.63178467	3.644599781	0.424972028	0.1840722
####	A_25_P00013607	hsa-miR-505-5p	-3.186611562	-22.95391967	7.203237427	-0.1871929	0.1840722
6747	A_25_P00015035	hsa-miR-320b	-3.180960888	-380.9638267	119.7637569	0.289177057	0.1840722
9987	A_25_P00012514	hsa-miR-181d	-3.180170591	-51.033509	16.04741241	0.239216165	0.1840722
####	A_25_P00010894	hsa-miR-361-5p	-3.179121426	-271.9679317	85.54814214	0.259922744	0.1840722
7208	A_25_P00015271	hsa-miR-320d	-3.176779925	-341.3042283	107.4371648	0.307921043	0.1840722

3343	A_25_P00013892	hsa-miR-199b-5p	-3.17616784	-45.81247638	14.423821	0.00278651	0.1840722
9338	A_25_P00012834	hsa-miR-652-3p	-3.172974361	-55.32060467	17.43493592	0.143141669	0.1840722
####	A_25_P00011716	ebv-miR-BART7	-3.172892505	-8.645141083	2.724687669	0.022384734	0.1840722
7854	A_25_P00012322	hsa-miR-371a-5p	-3.172517702	-89.49182667	28.20845621	0.190808213	0.1840722
4459	A_25_P00014898	hsa-miR-582-5p	-3.172038258	-15.04470743	4.742914874	-0.17219201	0.1840722
6931	A_25_P00014661	hsa-miR-550a-3p	-3.17071413	-47.97443367	15.13048219	0.080461534	0.1840722
####	A_25_P00010835	hsa-miR-197-3p	-3.168010063	-125.9588333	39.75960645	0.35804932	0.1840722
9052	A_25_P00012512	hsa-miR-193b-3p	-3.165220088	-151.1625967	47.7573731	0.327415533	0.1840722
6878	A_25_P00013090	hsa-miR-940	-3.162393346	-161.89286	51.19314465	0.368829648	0.1840722
988	A_25_P00012162	hsa-miR-128	-3.154576366	-1945.429883	616.7008364	0.246948149	0.1840722
7750	A_25_P00013090	hsa-miR-940	-3.15341345	-172.7816617	54.79194671	0.343963105	0.1840722
642	A_25_P00013371	hsa-miR-15b-3p	-3.152376217	-89.85733167	28.50463444	0.195787916	0.1840722
4940	A_25_P00011854	ebv-miR-BART13	-3.151878151	-99.62564133	31.60834162	0.117646615	0.1840722
3045	A_25_P00011007	hsa-miR-194-5p	-3.150853381	-56.671205	17.98598607	0.172373232	0.1840722
####	A_25_P00012323	hsa-miR-371a-5p	-3.148066422	-71.27897167	22.6421435	0.245559084	0.1840722
3704	A_25_P00013021	hsa-miR-760	-3.143942198	-18.12835865	5.766123391	-0.10227246	0.1840722
6560	A_25_P00013400	hsa-miR-132-5p	-3.142774755	-9.041758317	2.876998519	-0.21223366	0.1840722
####	A_25_P00015271	hsa-miR-320d	-3.142473915	-364.33483	115.9388558	0.301397493	0.1840722
5478	A_25_P00012161	hsa-miR-128	-3.141954485	-1236.848383	393.6557291	0.251506017	0.1840722
####	A_25_P00012704	hsa-miR-545-3p	-3.141929716	-9.25749525	2.946436135	-0.22767315	0.1840722
9508	A_25_P00013200	hsa-miR-26b-3p	-3.138234003	-13.2899125	4.234837965	0.171281342	0.1840722
9647	A_25_P00012196	hsa-miR-152	-3.136579875	-66.96502833	21.34969648	0.158024041	0.1840722
####	A_25_P00015271	hsa-miR-320d	-3.132127679	-363.543895	116.0693089	0.301321189	0.1840722
6919	A_25_P00013546	hsa-miR-340-3p	-3.131809867	-63.28256867	20.20638907	0.11730383	0.1840722
####	A_25_P00015034	hsa-miR-320b	-3.129025219	-148.5924983	47.48843104	0.290072336	0.1840722
####	A_25_P00010688	hsa-miR-498	-3.127690894	-18.25585533	5.836847679	0.275817442	0.1840722
8618	A_25_P00014832	hsa-miR-181a-5p	-3.12706271	-4750.187667	1519.057373	0.19513591	0.1840722
6050	A_25_P00012304	hsa-miR-361-3p	-3.126694009	-18.05744817	5.775252747	0.248746875	0.1840722
5730	A_25_P00013880	hsa-miR-129-5p	-3.125574794	-12.072523	3.862496915	-0.37980759	0.1840722
4691	A_25_P00013449	hsa-miR-149-3p	-3.125523637	-17.6238775	5.638695959	-0.23393446	0.1840722
8350	A_25_P00012162	hsa-miR-128	-3.124173576	-1982.056467	634.4258469	0.249040881	0.1840722
6578	A_25_P00012162	hsa-miR-128	-3.123403478	-2301.617283	736.8940002	0.252358845	0.1840722
8259	A_25_P00014832	hsa-miR-181a-5p	-3.118951905	-4412.395117	1414.704443	0.189579465	0.1840722
5456	A_25_P00013488	hsa-miR-30c-1-3p	-3.117563913	-17.143698	5.499068658	-0.12571232	0.1840722
####	A_25_P00015271	hsa-miR-320d	-3.117358795	-344.5321883	110.5205435	0.302189593	0.1840722

9588	A_25_P00014906	hsa-miR-1224-5p	-3.116051109	-78.49349517	25.19005382	0.204564639	0.1840722
4733	A_25_P00012625	hsa-miR-499a-5p	-3.115997724	-13.80124667	4.429158134	-0.16074603	0.1840722
####	A_25_P00012161	hsa-miR-128	-3.115897494	-1375.604233	441.4792964	0.248331765	0.1840722
5283	A_25_P00010881	hsa-miR-23b-3p	-3.115496081	-151.567145	48.64944171	0.268036619	0.1840722
6163	A_25_P00011853	ebv-miR-BART13	-3.110035162	-162.0164083	52.09471916	0.153414165	0.1840722
####	A_25_P00012654	hsa-miR-505-3p	-3.109853528	-137.4762583	44.20666668	0.157623151	0.1840722
5085	A_25_P00015035	hsa-miR-320b	-3.109618954	-314.48419	101.1327094	0.293979826	0.1840722
3732	A_25_P00012419	hsa-miR-423-5p	-3.108925289	-197.6966083	63.59001583	0.178521942	0.1840722
####	A_25_P00012161	hsa-miR-128	-3.108035425	-1330.389817	428.0484727	0.25283289	0.1840722
9580	A_25_P00012423	hsa-miR-423-3p	-3.107850463	-10.09792167	3.249165874	-0.29220868	0.1840722
2530	A_25_P00012162	hsa-miR-128	-3.107162644	-1825.080233	587.3784035	0.251980225	0.1840722
8384	A_25_P00015270	hsa-miR-320d	-3.107017707	-285.2144283	91.79684676	0.309224236	0.1840722
####	A_25_P00010894	hsa-miR-361-5p	-3.105518506	-230.6977967	74.28640217	0.272203459	0.1840722
####	A_25_P00012126	hsa-miR-222-3p	-3.104260073	-74.71948617	24.06998267	0.116749148	0.1840722
5292	A_25_P00014837	hsa-miR-27b-3p	-3.101159259	-189.503185	61.10720836	0.22688019	0.1840722
####	A_25_P00015270	hsa-miR-320d	-3.100628989	-243.182175	78.42994948	0.290899122	0.1840722
6658	A_25_P00014832	hsa-miR-181a-5p	-3.098173363	-4874.291667	1573.279186	0.199420817	0.1840722
####	A_25_P00014832	hsa-miR-181a-5p	-3.098098387	-4457.038833	1438.636956	0.195832118	0.1840722
5047	A_25_P00013089	hsa-miR-940	-3.097478943	-64.81966333	20.92658724	0.406983132	0.1840722
8559	A_25_P00015034	hsa-miR-320b	-3.096813259	-180.704645	58.35180551	0.270076343	0.1840722
821	A_25_P00013000	hsa-miR-887	-3.087597327	-32.21006599	10.43208119	-0.08205721	0.2604318
5259	A_25_P00015094	hsa-miR-548e	-3.085406915	-10.43397167	3.381716562	-0.27312575	0.2604318
6739	A_25_P00013366	hsa-let-7i-3p	-3.083067658	-15.174932	4.922023673	0.194671594	0.2604318
7115	A_25_P00015270	hsa-miR-320d	-3.082119965	-256.2263617	83.1331566	0.297339778	0.2604318
####	A_25_P00010926	hsa-miR-215	-3.082009235	-40.30036533	13.07600408	0.189700428	0.2604318
838	A_25_P00013089	hsa-miR-940	-3.073198693	-57.21383667	18.61703143	0.44580533	0.2604318
3475	A_25_P00012833	hsa-miR-652-3p	-3.0731156	-13.44012133	4.373451274	-0.28612219	0.2604318
3405	A_25_P00011230	hsa-miR-769-3p	-3.070311878	-15.55491233	5.066232016	-0.00154137	0.2604318
####	A_25_P00012161	hsa-miR-128	-3.070060348	-1284.517767	418.4014713	0.246065144	0.2604318
7500	A_25_P00012834	hsa-miR-652-3p	-3.069709693	-55.886346	18.20574308	0.17377122	0.2604318
6619	A_25_P00010761	hsa-miR-27b-3p	-3.069687429	-104.0197067	33.88609071	0.242738516	0.2604318
####	A_25_P00012512	hsa-miR-193b-3p	-3.063382311	-139.2381333	45.45241801	0.313956714	0.2604318
####	A_25_P00012243	hsa-miR-186-5p	-3.062733502	-175.798975	57.39937049	0.148023019	0.2604318
2625	A_25_P00012196	hsa-miR-152	-3.061754767	-68.98321367	22.53061362	0.144869375	0.2604318
####	A_25_P00012243	hsa-miR-186-5p	-3.061679874	-185.13654	60.46894111	0.157702634	0.2604318

6784	A_25_P00010287	hsa-miR-181c-5p	-3.060485587	-38.16065617	12.46882401	0.191211253	0.2604318
####	A_25_P00014161	hsa-miR-501-5p	-3.052886231	-15.80009067	5.17546003	0.042677321	0.2604318
5890	A_25_P00010815	hsa-miR-30c-5p	-3.052292311	-203.9778867	66.82776938	0.168883227	0.2604318
####	A_25_P00013313	hsa-miR-181a-2-3p	-3.050777224	-25.97503133	8.514234053	0.08242197	0.2604318
####	A_25_P00013981	hsa-miR-361-5p	-3.045942334	-137.6675283	45.19702385	0.249696002	0.2604318
8991	A_25_P00014820	hsa-miR-23a-3p	-3.045183326	-621.720765	204.1652993	0.151042175	0.2604318
3431	A_25_P00012162	hsa-miR-128	-3.043380218	-1897.123333	623.3606047	0.250670844	0.2604318
####	A_25_P00011853	ebv-miR-BART13	-3.043093532	-162.8376383	53.51055978	0.151440628	0.2604318
####	A_25_P00013142	hsa-miR-15a-3p	-3.042028244	-35.5255405	11.67824151	0.155448575	0.2604318
3053	A_25_P00011007	hsa-miR-194-5p	-3.041446865	-55.61821	18.28676037	0.227282528	0.2604318
3167	A_25_P00013852	hsa-miR-30a-5p	-3.04101124	-12.21875883	4.017991999	-0.72049121	0.2604318
5918	A_25_P00015274	hsa-miR-1826_v15.0	-3.040619037	-23.9907015	7.890071465	-0.01823394	0.2604318
2772	A_25_P00013162	hsa-miR-19b-1-5p	-3.03866937	-71.65725817	23.58178842	0.125211368	0.2604318
8341	A_25_P00010894	hsa-miR-361-5p	-3.038209902	-243.3331917	80.09097445	0.260929218	0.2604318
####	A_25_P00015003	hsa-miR-1226-5p	-3.03756918	-39.90080683	13.13576892	0.201753223	0.2604318
9664	A_25_P00010894	hsa-miR-361-5p	-3.037397421	-248.5961433	81.8451157	0.261776223	0.2604318
####	A_25_P00011799	hsv1-miR-H1_v14.0	-3.036982653	-13.68668967	4.506673639	0.237564977	0.2604318
5404	A_25_P00015035	hsa-miR-320b	-3.036966565	-319.377485	105.1633194	0.286305223	0.2604318
####	A_25_P00010470	hsa-miR-10a-5p	-3.03692471	-7.870212017	2.591507123	-0.15443714	0.2604318
####	A_25_P00014925	hsa-miR-1225-3p	-3.03596032	-18.14810667	5.97771537	0.473375916	0.2604318
434	A_25_P00013981	hsa-miR-361-5p	-3.035436141	-153.6058233	50.60420189	0.240135	0.2604318
7917	A_25_P00013689	kshv-miR-K12-3	-3.027565976	-182.6676117	60.33480794	0.212086254	0.2604318
6591	A_25_P00012354	hsa-miR-342-5p	-3.02675791	-17.02636283	5.625280693	0.231737327	0.2604318
9079	A_25_P00012831	hsa-miR-636	-3.02665728	-13.89535767	4.590991441	0.029631206	0.2604318
4408	A_25_P00010894	hsa-miR-361-5p	-3.02375153	-235.504105	77.88474109	0.27395273	0.2604318
####	A_25_P00013090	hsa-miR-940	-3.021778947	-172.923555	57.22574617	0.354667499	0.2604318
2940	A_25_P00014832	hsa-miR-181a-5p	-3.019449294	-4300.0905	1424.130721	0.18952311	0.2604318
3223	A_25_P00012419	hsa-miR-423-5p	-3.012378038	-209.360365	69.50003032	0.173051197	0.2604318
9768	A_25_P00014852	hsa-miR-365a-3p	-3.011712526	-179.6354033	59.64560089	0.288072008	0.2604318
2486	A_25_P00013090	hsa-miR-940	-3.011497704	-162.6094767	53.99621473	0.362157359	0.2604318
9466	A_25_P00010882	hsa-miR-23b-3p	-3.00914313	-59.86878833	19.89562667	0.231322099	0.2604318
1841	A_25_P00012161	hsa-miR-128	-3.008759122	-1305.244717	433.8149594	0.251415324	0.2604318
2505	A_25_P00015098	hsa-miR-1285-3p	-3.005120185	-13.78990867	4.58880438	-4.4396481	0.2604318
6930	A_25_P00010894	hsa-miR-361-5p	-3.003834298	-283.66993	94.4359448	0.258491706	0.2604318
8657	A_25_P00012305	hsa-miR-361-3p	-3.002829001	-40.956266	13.63922687	0.228879259	0.2604318

4436	A_25_P00012654	hsa-miR-505-3p	-2.995496273	-134.6137433	44.93871167	0.159894142	0.2604318
####	A_25_P00013546	hsa-miR-340-3p	-2.993515553	-64.09031067	21.40971361	0.102910416	0.2604318
####	A_25_P00012196	hsa-miR-152	-2.991578765	-69.15251667	23.11572654	0.150876352	0.2604318
4804	A_25_P00012305	hsa-miR-361-3p	-2.990695076	-45.62611833	15.25602483	0.204666984	0.2604318
5660	A_25_P00014847	hsa-miR-150-5p	-2.990675003	-128.4305617	42.94367042	0.202491136	0.2604318
####	A_25_P00012243	hsa-miR-186-5p	-2.990595552	-185.2752733	61.95263456	0.138479072	0.2604318
####	A_25_P00015270	hsa-miR-320d	-2.990269667	-269.2650283	90.04707211	0.295541938	0.2604318
1601	A_25_P00012955	hsa-miR-875-5p	-2.989560391	-29.14348283	9.748417499	-0.12352171	0.2604318
####	A_25_P00013089	hsa-miR-940	-2.988785453	-66.070365	22.1060916	0.405706722	0.2604318
5864	A_25_P00015124	hsa-miR-1300_v13.0	-2.988550327	-16.14836167	5.403409647	-0.03044393	0.2604318
7515	A_25_P00013689	kshv-miR-K12-3	-2.986028445	-180.3108333	60.38483446	0.233422934	0.2604318
####	A_25_P00012514	hsa-miR-181d	-2.984167754	-46.54000367	15.59563922	0.296669381	0.2604318
877	A_25_P00014897	hsa-miR-582-5p	-2.981449941	-6.553779093	2.198185186	0.068810759	0.2604318
7633	A_25_P00012324	hsa-miR-371a-5p	-2.980200225	-70.95946667	23.81030176	0.240899101	0.2604318
8184	A_25_P00010130	hsa-miR-497-5p	-2.978223104	-39.45166267	13.24671164	0.115119235	0.2604318
4178	A_25_P00012161	hsa-miR-128	-2.975075686	-1204.5144	404.8684898	0.255557204	0.2604318
####	A_25_P00013354	hsa-miR-223-5p	-2.972621081	-18.65596633	6.275931517	0.039448138	0.2604318
614	A_25_P00010635	hsa-miR-584-5p	-2.972065321	-18.15058835	6.107062392	0.195796997	0.2604318
3474	A_25_P00010882	hsa-miR-23b-3p	-2.971054927	-54.975435	18.50367508	0.283748376	0.2604318
8974	A_25_P00011985	hsa-let-7e-5p	-2.970796939	-16.96051683	5.709079812	0.085089307	0.2604318
5400	A_25_P00013883	hsa-miR-30c-5p	-2.969358515	-712.0458167	239.7978597	0.196588787	0.2604318
1346	A_25_P00012215	hsa-miR-126-3p	-2.966746886	-36.31558667	12.24087799	0.042363933	0.2604318
1777	A_25_P00012830	hsa-miR-636	-2.965573795	-19.47112583	6.565719547	-0.05182732	0.2604318
4475	A_25_P00013209	hsa-miR-29a-5p	-2.963725399	-33.16930983	11.19176218	0.057488796	0.2604318
2292	A_25_P00015210	hsa-miR-1275	-2.962198881	-185.3631033	62.57618438	0.39335191	0.2604318
####	A_25_P00010130	hsa-miR-497-5p	-2.962072458	-42.57500407	14.37338373	0.073653062	0.2604318
2088	A_25_P00013023	hsa-miR-760	-2.960889687	-19.90028387	6.721048729	0.201669869	0.2604318
1988	A_25_P00013893	hsa-miR-199b-5p	-2.960077137	-74.80204767	25.27030351	0.086411269	0.2604318
####	A_25_P00013020	hsa-miR-760	-2.959067592	-21.34048333	7.211894515	-0.07724761	0.2604318
5428	A_25_P00015003	hsa-miR-1226-5p	-2.958541509	-45.0840042	15.23859106	0.087640197	0.2604318
####	A_25_P00012243	hsa-miR-186-5p	-2.958390657	-178.3841417	60.29769639	0.158977021	0.2604318
####	A_25_P00015142	hsa-miR-1246	-2.955556364	-75.666225	25.60134732	0.380526378	0.2604318
####	A_25_P00014178	hsa-miR-532-5p	-2.954752945	-60.92961	20.62088138	0.196193391	0.2604318
5294	A_25_P00015034	hsa-miR-320b	-2.952835554	-169.34816	57.35102986	0.265876021	0.2604318
8429	A_25_P00014660	hsa-miR-550a-3p	-2.94952499	-31.265441	10.60016142	0.062891039	0.2604318

####	A_25_P00012837	hsa-miR-548d-5p	-2.945875705	-9.3394165		3.170336237	0.393270961	0.2604318
5342	A_25_P00012815	hsa-miR-628-5p	-2.943648493	-9.042863667		3.071991676	-0.43476036	0.2604318
4245	A_25_P00014182	hsa-miR-455-5p	-2.939257064	-18.01098367		6.127733395	0.130492943	0.2604318
3322	A_25_P00013098	hsa-miR-942	-2.938812468	-11.40397983		3.880472115	0.213417371	0.2604318
####	A_25_P00013689	kshv-miR-K12-3	-2.935644773	-176.102005		59.98750484	0.238637167	0.2604318
####	A_25_P00011005	hsa-miR-103a-3p	-2.933514775	-1209.875517		412.4320515	0.19060376	0.2604318
8578	A_25_P00013271	hsa-miR-16-2-3p	-2.932940692	-197.71299		67.41117901	0.207251468	0.2604318
####	A_25_P00013090	hsa-miR-940	-2.932390073	-173.8871767		59.29878779	0.341752262	0.2604318
6450	A_25_P00013892	hsa-miR-199b-5p	-2.930524321	-36.20822756		12.35554583	0.13989615	0.2604318
5932	A_25_P00010635	hsa-miR-584-5p	-2.928479065	-15.5869425		5.322538477	0.325679914	0.2604318
####	A_25_P00015210	hsa-miR-1275	-2.925324489	-183.85629		62.84987894	0.402525491	0.2604318
####	A_25_P00012954	hsa-miR-875-5p	-2.925116757	-24.67727765		8.436339366	-0.05857907	0.2604318
####	A_25_P00012418	hsa-miR-423-5p	-2.921676399	-88.36601567		30.24497021	0.164869342	0.2604318
1439	A_25_P00013235	hsa-miR-93-3p	-2.920034049	-30.01219683		10.27802975	0.142567784	0.2604318
####	A_25_P00012419	hsa-miR-423-5p	-2.919654435	-183.1557017		62.73197933	0.190369931	0.2604318
2046	A_25_P00014852	hsa-miR-365a-3p	-2.917076206	-156.02671		53.48736165	0.298641936	0.2604318
6703	A_25_P00010344	hsa-miR-557	-2.915202549	-19.223085		6.594082117	0.15408478	0.2604318
####	A_25_P00012692	hsa-miR-532-3p	-2.914639653	-38.90160467		13.34696885	0.194479631	0.2604318
9552	A_25_P00014820	hsa-miR-23a-3p	-2.914141866	-664.6105833		228.0639083	0.150939011	0.2604318
####	A_25_P00010881	hsa-miR-23b-3p	-2.913972125	-142.9368		49.0522194	0.281699717	0.2604318
2391	A_25_P00013981	hsa-miR-361-5p	-2.910012624	-143.0855367		49.1700742	0.27108434	0.2604318
6347	A_25_P00014866	hsa-miR-497-5p	-2.908683331	-107.4017583		36.92452773	0.186669076	0.2604318
7641	A_25_P00015270	hsa-miR-320d	-2.905330379	-283.137175		97.45438147	0.303026366	0.2604318
####	A_25_P00011004	hsa-miR-103a-3p	-2.904124412	-1876.501917		646.1506639	0.179683008	0.2604318
####	A_25_P00013277	hsa-miR-129-1-3p	-2.897779903	-28.23843167		9.744850406	0.427776863	0.2604318
3683	A_25_P00013883	hsa-miR-30c-5p	-2.895756138	-637.2263667		220.0552589	0.197123279	0.2604318
4012	A_25_P00014610	hsa-miR-30e-3p	-2.895284895	-82.74963667		28.58082699	0.146271429	0.2604318
2884	A_25_P00010676	hsa-miR-24-3p	-2.894990879	-214.1167183		73.96110291	0.17218107	0.2604318
####	A_25_P00010640	hsa-miR-601	-2.894756486	-24.13991433		8.339186543	0.261148485	0.2604318
7823	A_25_P00011853	ebv-miR-BART13	-2.893339675	-168.74508		58.32190442	0.16011581	0.2604318
1306	A_25_P00010761	hsa-miR-27b-3p	-2.893244474	-94.019455		32.49620136	0.256803093	0.2604318
####	A_25_P00014906	hsa-miR-1224-5p	-2.893226805	-79.2700095		27.39847749	0.20063899	0.2604318

Estimated Miss rates for Delta=1.42112278626184

Quantiles	Cutpoints	Miss Rate(%)
0 -> 0.05	-2.892 -> -2.175	75.26
0.05 -> 0.1	-2.175 -> -1.868	72.97
0.1 -> 0.15	-1.868 -> -1.606	64.57
0.15 -> 0.2	-1.606 -> -1.296	26.68
0.2 -> 0.25	-1.296 -> -1.028	0
0.25 -> 0.75	-1.028 -> 0.203	8.89
0.75 -> 0.8	0.203 -> 0.343	0
0.8 -> 0.85	0.343 -> 0.482	0
0.85 -> 0.9	0.482 -> 0.626	0
0.9 -> 0.95	0.626 -> 0.837	0
0.95 -> 1	0.837 -> 15.522	0

Reh cell line: shSCR versus shRNA for SHOC2

Input parameters

Data type?	Two class unpaired
Array or Seq data?	array
Arrays centered?	FALSE
Delta	25.32162178
Minimum fold change	2
Test statistic	standard
Are data are log scale?	FALSE
Number of permutations	100
Input percentile for exchangeability factor s0	Automatic choice
Number of neighbors for KNN	10
Seed for Random number generator	83016168

Computed values

Estimate of pi0 (proportion of null genes)	0.414
s0 percentile	0
False Discovery Rate (%)	0.213483381

List of Significant Genes for Delta = 25.322

Positive genes (15)

Row	Probe ID	Gene ID	Score(d)	Numerator(r)	Denominator(s+s	Fold Change	q-value(%)
3845	A_33_P336:	NR_003008	200.5246209	2651.62035	13.22341535	6.674559471	0
42678	A_23_P703!	NM_001025370	197.7436772	1764.6925	8.924141215	2.693895299	0
42536	A_23_P103!	NM_178134	110.6820105	134.5707735	1.215832391	19.95596219	0.243622211
6821	A_33_P330!	AK123993	82.32099874	845.3113	10.26847722	3.30657689	0.243622211
18195	A_23_P505!	NM_004823	77.17410175	509.1768	6.597767754	2.753078097	0.243622211
42000	A_33_P334(	NP105946	76.94179292	454.28835	5.904311984	2.601899444	0.243622211
27986	A_23_P348:	NM_005253	75.85120087	30.735115	0.405202748	6.400311173	0.243622211
5073	A_33_P334(	NM_032968	68.20095812	310.657895	4.555037107	23.88589976	0.243622211
43403	A_33_P342:	A_33_P3423845	67.35720191	987.01175	14.65339595	2.036257559	0.243622211
4931	A_33_P337	AK094630	66.25828645	185.359675	2.797531976	18.41451619	0.243622211

33164	A_33_P337	A_33_P3373200	64.76855748	355.695215	5.491788436	13.75874844	0.243622211
16444	A_33_P321	AJ298317	62.95404353	500.046555	7.943041097	11.23801381	0.243622211
13310	A_33_P341	ENST0000042105	62.52884703	26524.285	424.1927727	2.763535877	0.243622211
43173	A_23_P121	NM_000549	60.84534486	435.71585	7.161038383	4.875163701	0.243622211
33980	A_23_P134	NM_002347	60.51680613	164.709682	2.721718024	27.182554	0.243622211

Negative genes (82)

Row	Probe ID	Gene ID	Score(d)	Numerator(r)	Denominator(s+s	Fold Change	q-value(%)
16202	A_23_P657	NM_014106	-612.4238255	-386.676405	0.63138694	0.188240408	0
4831	A_33_P328	NR_024398	-478.7247436	-290.50968	0.606840745	0.071053769	0
15245	A_33_P323	NM_033225	-450.2698508	-60.7529905	0.134925735	0.113207658	0
3837	A_23_P173	NM_024034	-358.8636344	-426.619835	1.188807653	0.018958143	0
5194	A_33_P328	NM_031453	-203.190685	-8404.587	41.36305264	0.133100296	0
31204	A_33_P327	A_33_P3279526	-199.7197533	-174.096654	0.871704732	0.038689467	0
5583	A_23_P104	NM_004183	-187.6957063	-179.375085	0.955669624	0.128798661	0
18548	A_32_P959	NM_001004419	-183.7868425	-248.833615	1.353925078	0.057337022	0
41435	A_23_P106	NM_001038640	-173.6028972	-322.288085	1.856467203	0.18039048	0
13042	A_33_P342	NM_001130858	-168.1815261	-42.379188	0.251984799	0.187449594	0
15407	A_23_P653	NM_138433	-162.6212253	-355.61845	2.186789882	0.031430176	0
41193	A_23_P994	NM_000059	-157.6403015	-252.393115	1.60106973	0.25499812	0
26990	A_33_P380	NM_001036	-153.0516965	-76.95865	0.502827814	0.182463123	0
12246	A_33_P325	ENST0000037892	-148.5995905	-21.0208025	0.141459357	0.264665335	0
9499	A_33_P331	NM_002874	-138.5948841	-337.22205	2.433149334	0.476828606	0
27883	A_33_P321	NM_024529	-126.6875979	-352.828885	2.785030981	0.171575132	0
23251	A_33_P341	AK055900	-98.13682107	-45.265734	0.461251277	0.145053307	0
40887	A_24_P417	NM_001029880	-97.93899929	-94.26272	0.962463581	0.417499777	0
27955	A_33_P332	NM_006954	-85.87552755	-849.0394	9.886860952	0.377649795	0
40437	A_33_P323	A_33_P3233000	-84.02700247	-607.05565	7.224530593	0.341504992	0
38733	A_33_P337	NM_001040284	-81.69646045	-45.4637815	0.556496343	0.16497527	0
14520	A_33_P350	AK091571	-80.29828371	-306.39325	3.815688653	0.392478103	0
22976	A_33_P380	NM_133372	-80.12317646	-477.77155	5.962963166	0.230577508	0
823	A_23_P599	NM_054028	-78.58435713	-285.039995	3.627184918	0.073349851	0
41135	A_23_P105	NM_020128	-73.26988815	-150.8398	2.058687461	0.491172245	0
41027	A_33_P385	NM_018328	-72.81733166	-158.883105	2.181940774	0.091392762	0

2953	A_23_P377:	NM_001192	-71.17678172	-26.758517	0.375944463	0.245264179	0
6535	A_23_P320:	NM_152446	-70.10768578	-302.0141	4.307860067	0.293276956	0
24609	A_23_P319:	NM_000050	-69.58705296	-1257.94191	18.07724076	0.018136887	0
41268	A_23_P408:	NM_015642	-69.51246262	-103.094843	1.483113087	0.079031756	0
15795	A_33_P328:	NM_001201407	-68.38176204	-76.206465	1.114426753	0.364628969	0
37747	A_33_P335:	NM_000959	-65.77150144	-269.282755	4.094216326	0.142278853	0
2316	A_23_P106:	NM_018659	-65.15132029	-67.506777	1.03615363	0.118846682	0
22784	A_33_P320:	AK097937	-64.18806867	-108.715725	1.693706124	0.181971937	0
16727	A_33_P335:	A_33_P3356941	-64.05655882	-56.7734125	0.886301318	0.112941418	0
31144	A_23_P100:	NM_182538	-63.26820917	-906.874825	14.33381531	0.063878541	0
14842	A_33_P326:	THC2674845	-62.91526469	-169.367935	2.692000675	0.294811973	0
30464	A_32_P147:	NM_178504	-61.64340893	-45.7040505	0.741426396	0.142219499	0
41229	A_33_P331:	NM_001005851	-61.6003867	-111.761186	1.814293578	0.072541674	0
9471	A_23_P361:	NM_152680	-61.56734129	-142.9111845	2.32121741	0.058045716	0
1794	A_33_P321:	BC020894	-60.18611455	-110.6076035	1.837759495	0.074705273	0
41521	A_33_P340:	BC066931	-60.12904642	-66.214217	1.101201847	0.138061246	0
27879	A_23_P322:	NM_005802	-59.97471435	-1611.1824	26.86436138	0.26823327	0
39104	A_33_P332:	NM_015713	-59.02524885	-62.283785	1.055205801	0.30301399	0
38138	A_23_P432:	NM_176816	-58.20578828	-1181.416185	20.2972285	0.068869027	0
40395	A_33_P321:	NM_152279	-57.68352565	-52.604685	0.911953359	0.315924223	0
733	A_33_P325:	ENST0000041916(	-57.59327831	-110.553665	1.919558467	0.229631448	0
22596	A_23_P625:	NM_138705	-57.51221862	-2397.44405	41.68582098	0.047343636	0
16090	A_23_P112:	NM_004606	-56.12241362	-320.0021	5.701859193	0.243795263	0
43379	A_33_P340:	THC2560329	-54.87035692	-651.71812	11.87741718	0.083338398	0.383479407
12274	A_33_P338:	NR_024549	-54.85463561	-611.46715	11.14704606	0.332508303	0.383479407
37346	A_33_P341:	ENST0000053519(	-54.63019713	-496.1722	9.08237982	0.087427655	0.383479407
30576	A_33_P321:	NM_015330	-53.29650269	-133.28264	2.500776473	0.167843222	0.383479407
22523	A_33_P339:	NM_003921	-51.5106828	-96.3421	1.87033242	0.307187432	0.383479407
33667	A_33_P337:	A_33_P3375496	-50.5293999	-114.0755	2.257606467	0.480464385	0.383479407
10392	A_24_P415:	NM_015978	-49.49017039	-135.010863	2.728033909	0.072449197	0.383479407
30465	A_24_P252:	NM_001039656	-49.45655988	-78.139749	1.579967333	0.099677155	0.383479407
41825	A_33_P339:	NM_004614	-49.26120188	-58.4577525	1.18668953	0.136070921	0.383479407
17093	A_33_P330:	NM_000965	-49.0938253	-106.5145355	2.169611654	0.062373285	0.383479407
43859	A_33_P337:	AK092544	-47.77911407	-34.1814785	0.715406285	0.225764691	0.383479407
39563	A_33_P339:	NM_032530	-47.29111303	-233.69608	4.94164897	0.180255795	0.383479407

27876	A_23_P596:	NM_021930	-46.95803211	-1116.43395	23.77514346	0.32031209	0.383479407
27821	A_24_P100(	NM_052852	-46.67168228	-913.7221	19.57765513	0.189525107	0.383479407
28794	A_32_P218!	NM_004559	-45.45608722	-8682.0375	190.9983466	0.493683463	0.383479407
17729	A_23_P313:	NM_018334	-45.11847879	-92.07031	2.040634181	0.278058284	0.383479407
43087	A_33_P326:	NM_017661	-44.96912398	-45.84092	1.019386547	0.336106116	0.383479407
23970	A_33_P334:	THC2723408	-44.90071784	-22.179204	0.493961012	0.2469113	0.383479407
28218	A_23_P503(	NM_206818	-44.45070723	-3439.53865	77.37871598	0.120597437	0.383479407
24952	A_24_P117:	NM_145297	-44.01842721	-3505.303	79.63262711	0.312884209	0.383479407
13058	A_23_P104!	NM_004183	-43.78636784	-205.46653	4.692477137	0.111600005	0.383479407
10796	A_33_P335:	NM_014351	-42.74506825	-168.829195	3.949676581	0.183225368	0.383479407
8250	A_33_P328:	NM_022780	-42.72810379	-319.46885	7.476785105	0.421464999	0.383479407
34069	A_32_P925!	THC2771198	-42.69498924	-88.6282305	2.075846184	0.082864002	0.383479407
29238	A_32_P539!	NR_015392	-42.44920596	-30.2846325	0.71343225	0.207069084	0.383479407
22825	A_33_P331(	NM_001113523	-42.37083365	-1446.78652	34.145812	0.047335062	0.383479407
23560	A_33_P328(	A_33_P3280502	-41.92860515	-2664.26535	63.54290443	0.150823371	0.383479407
14825	A_32_P220:	NM_033160	-41.82952144	-93.385755	2.232532235	0.386775074	0.383479407
18468	A_23_P115(	NM_024749	-41.73792976	-733.42435	17.57213053	0.328645711	0.383479407
20519	A_32_P837!	NM_015230	-41.48886623	-1649.85887	39.76630407	0.069148246	0.383479407
35465	A_33_P333:	NM_001001433	-41.12734028	-757.6573	18.42222947	0.310825928	0.383479407
26436	A_33_P327:	THC2750292	-41.12053519	-1913.05215	46.52303627	0.097348244	0.383479407
38437	A_33_P336:	NM_003161	-40.93913972	-187.12497	4.570808554	0.248890616	0.383479407

Estimated Miss rates for Delta=25.3216217849966

Quantiles	Cutpoints	Miss Rate(%)
0 ->0.05	-39.894 ->-9.34:	91.67
0.05 ->0.1	-9.342 ->-6.484	91.63
0.1 ->0.15	-6.484 ->-4.709	90.69
0.15 ->0.2	-4.709 ->-3.466	88.26
0.2 ->0.25	-3.466 ->-2.538	83.76
0.25 ->0.75	-2.538 ->1.261	36.13
0.75 ->0.8	1.261 ->1.402	80.89
0.8 ->0.85	1.402 ->1.638	68.6
0.85 ->0.9	1.638 ->2.272	74.27
0.9 ->0.95	2.272 ->3.281	79.72

0.95 -> 1	3.281 -> 59.677	59.48
